# Essential Regulation of Spermatogonial Stem Cell Fate Decisions and Male Fertility by APBB1 via Interaction with KAT5 and GDF15 in Humans and Mice

**DOI:** 10.34133/research.0647

**Published:** 2025-03-27

**Authors:** Dai Zhou, Bang Liu, Lvjun Liu, Guangmin Liu, Fang Zhu, Zenghui Huang, Shusheng Zhang, Zuping He, Liqing Fan

**Affiliations:** ^1^ Hunan Provincial Key Laboratory of Regional Hereditary Birth Defect Prevention and Control, Changsha Hospital for Maternal and Child Health Care Affiliated to Hunan Normal University, Changsha, Hunan 410000, China.; ^2^ Institute of Reproduction and Stem Cell Engineering, School of Basic Medicine Science, Central South University, Changsha, Hunan 410000, China.; ^3^ Key Laboratory of Model Animals and Stem Cell Biology in Hunan Province; Engineering Research Center of Reproduction and Translational Medicine of Hunan Province, Institute of Interdisciplinary Studies, Hunan Normal University, Hunan 410013, China.; ^4^ Hainan Academy of Medical Sciences, Hainan Medical University, Hainan 570311, China.; ^5^ Reproductive and Genetic Hospital of CITIC-Xiangya, Changsha, Hunan 410000, China.

## Abstract

Spermatogonial stem cells (SSCs) are essential for initiating and maintaining normal spermatogenesis, and notably, they have important applications in both reproduction and regenerative medicine. Nevertheless, the molecular mechanisms controlling the fate determinations of human SSCs remain elusive. In this study, we identified a selective expression of APBB1 in dormant human SSCs. We demonstrated for the first time that APBB1 interacted with KAT5, which led to the suppression of GDF15 expression and consequent inhibition of human SSC proliferation. Intriguingly, Apbb1^−/−^ mice assumed the disrupted spermatogenesis and markedly reduced fertility. SSC transplantation assays revealed that Apbb1 silencing enhanced SSC colonization and impeded their differentiation, which resulted in the impaired spermatogenesis. Notably, 4 deleterious *APBB1* mutation sites were identified in 2,047 patients with non-obstructive azoospermia (NOA), and patients with the c.1940C>G mutation had a similar testicular phenotype with Apbb1^−/−^ mice. Additionally, we observed lower expression levels of APBB1 in NOA patients with spermatogenic arrest than in obstructive azoospermia patients with normal spermatogenesis. Collectively, our findings highlight an essential role of APBB1/KAT5/GDF15 in governing human SSC fate decisions and maintaining normal spermatogenesis and underscore them as therapeutic targets for treating male infertility.

## Introduction

Infertility has been recognized as a global health concern, and it affects approximately 15% of couples of reproductive age worldwide [1]. Approximately half of the infertile cases are attributed to male factors [2]. Non-obstructive azoospermia (NOA) represents the most severe form of male infertility [3]. While a minority of NOA patients may have their genetic children through microsurgical testicular sperm extraction and the assisted reproductive technologies, there is not yet effective solution for these patients to meet their reproduction requirement [4]. Spermatogonial stem cells (SSCs) are essential for initialing and maintaining normal spermatogenesis [5]. Nevertheless, due to the differences in cell types and phenotypes of SSCs between rodents and humans SSC classification [6], the regulatory mechanisms governing human SSCs remain largely elusive. As a result, the regulatory mechanisms governing human SSCs remain largely unknown. Therefore, it is crucial to uncover the molecular mechanisms underlying the fate decisions of human SSCs for their applications in reproductive and regenerative medicine because of remarkable plasticity of these cells.

The fate determinations of SSCs in vivo include self-renewal to retain stem cell pool, differentiation into spermatocytes, and apoptosis to maintain the numbers of male germ cells, which are regulated by genetic and epigenetic factors. Glial cell line-derived neurotrophic factor (GDNF) has been shown to play a crucial role in the self-renewal of mouse SSCs [7,8]. The combination of GDNF with fibroblast growth factor 2 (FGF2) and leukemia inhibitory factor (LIF) has enabled the long-term culture of mouse SSCs [9]. Furthermore, other growth factors, including fibroblast growth factor 9 (FGF9) [10], vascular endothelial growth factor (VEGF) [11], and Wnt [12], have been demonstrated to enhance SSC self-renewal. However, Wnt proteins have also been found to induce SSC differentiation in mice [13]. Several intrinsic factors, including Plzf [14], Foxo1 [15], Nedd4 [16], Carf [17], and Spocd1 [18], have been reported to be involved in SSC self-renewal. On the other hand, several molecules, including retinoic acid (RA) [19], bone morphogenetic protein 4 (Bmp4) [20], and Stra8 [21], have been identified to be associated with SSC differentiation.

Research on the regulation of human SSCs is still in its infancy, due to the limited availability of human testicular tissues and lacking of long-term culture and expansion of human SSCs in vitro. FGF5 has been shown to stimulate human SSC proliferation by activating AKT and extracellular signal–regulated kinase (ERK) pathways [22]. Recently, we have demonstrated that OIP5 interaction with NCK2 is required for regulating the self-renewal and apoptosis of SSCs via modulation of cell cyclins and cell cycle progression [23]. Despite these findings, the underlying regulatory mechanisms of human SSCs remain largely unknown.

The advent of single-cell RNA sequencing (scRNA-seq) technology enables the analysis of cellular development dynamics and RNA expression profiles at the individual cell level. This technology facilitates the identification of SSCs from complex germ cell populations and allows for the characterization of SSC-specific transcriptional profiles. By integrating 6 testis single-cell sequencing data, we identified a number of genes that are differentially expressed in SSCs, including *APBB1* (amyloid β precursor protein binding family B member 1), *ASB9* [24], and TCF3 [25]. APBB1 has a distinctive expression pattern, since it is only expressed in SSCs at early developmental stages and its level is decreased as differentiation progresses, implicating that APBB1 may be involved in regulating the fate determinations of SSCs.

APBB1 is a scaffolding protein first identified in the mouse brain, and it is involved in neural development [26]. This protein has been shown to interact with signaling proteins and transcription factors that control cell proliferation [27]. APBB1 enhances the interaction of the E3 ubiquitin ligase Itch with Notch1 to increase the degradation of Notch1 [27] and control neuronal cell differentiation [28]. Knockdown of the 97-kDa isoform of APBB1 results in abnormal developmental dynamics of Gn-RH1 neurons in mice, with a 25% increase in the number of Gn-RH1 neurons and the prolonged neurogenesis [28]. Notably, APBB1 interacts with TAG1-APP signaling to inhibit neurogenesis [29]. The APP intracellular structural domain (AICD) of APBB1 forms a transcriptional regulatory complex with KAT5, which regulates the expression of stathmin and KAI1 to affect neurogenesis [30]. However, the role of this complex in controlling KAI1 is controversial. APBB1 can interact with the nucleosome assembly factor SET through its WW structural domain to activate KAI1 transcription [30]. On the other hand, APBB1 down-regulates KAI1 expression by interacting with estrogen receptor α [31]. These findings highlight the pivotal roles of APBB1 in neurogenesis and cell fate regulation. Nevertheless, the function and mechanisms of APBB1 in regulating SSC fate determinations remain to be explored.

In this study, we explored the specific expression of APBB1 in human SSCs through scRNA-seq analysis and immunohistochemistry. APBB1 silencing stimulated cellular proliferation of human SSCs and suppressed their apoptosis. Intriguingly, we uncovered a novel interaction between APBB1 and KAT5, which modulated the MAPK (mitogen-activated protein kinase) and WNT signaling pathways and repressed the expression of growth differentiation factor 15 (GDF15). Furthermore, our conditional knockout of *Apbb1* in mouse testes led to the disrupted spermatogenesis and a notable decrease in fertility. SSC transplantation indicated that the silencing of Apbb1 augmented SSC colonization and hindered their differentiation. Notably, a significantly lower level of APBB1 was observed in patients with NOA compared to obstructive azoospermia (OA) patients with normal spermatogenesis. Collectively, our findings delineate the roles and regulatory mechanisms of APBB1 in the fate decisions of human SSCs. This study is thus of unusual importance since it provides novel insights into the molecular mechanisms of human spermatogenesis and may offer new targets for gene therapy of male infertility.

## Results

### Single-cell transcriptomic analysis of normal human testicular cells

**Fig. 1.**
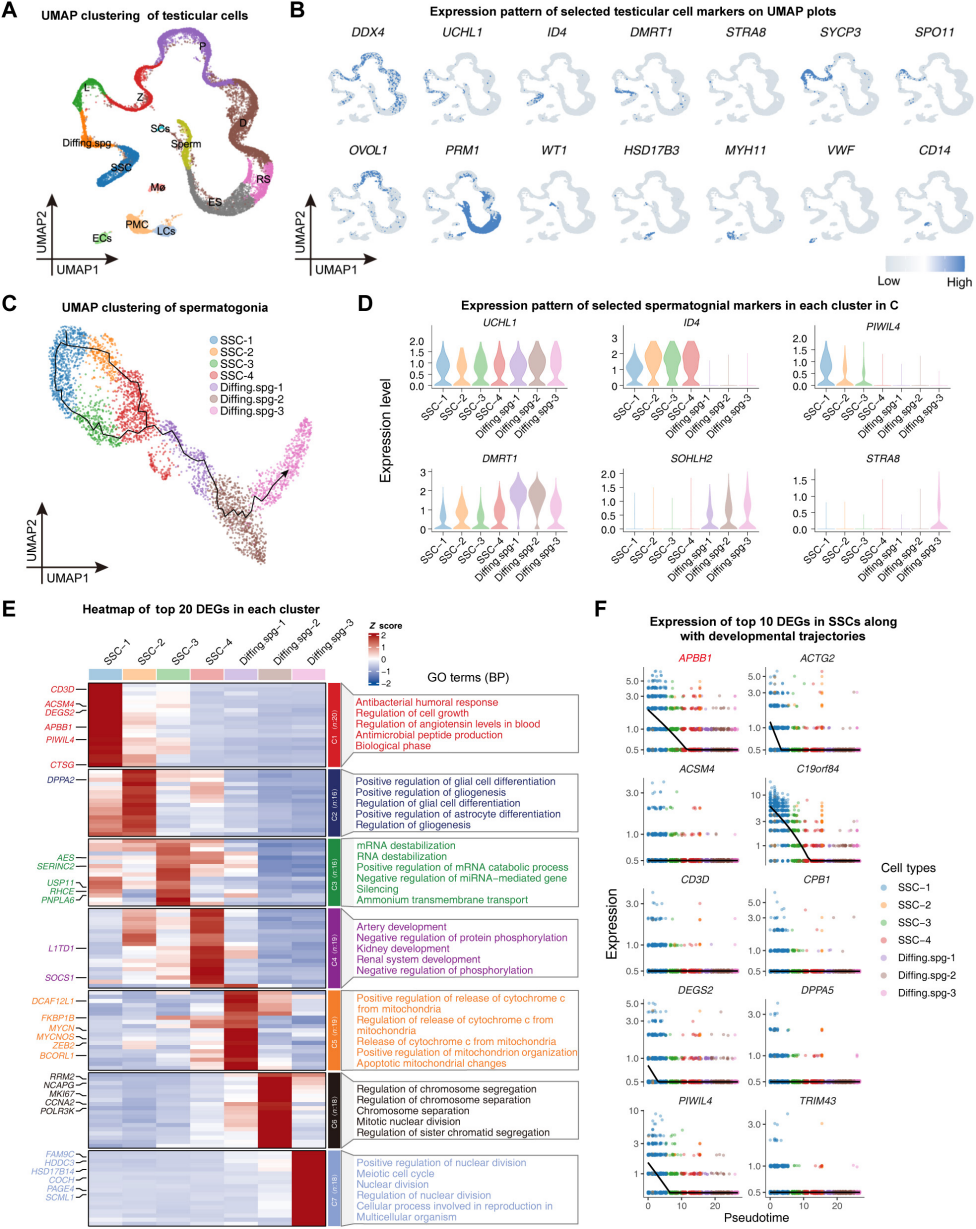
Single-cell transcriptome profiles of testicular cells from normal adult men. (A) UMAP plot showed the annotated testicular cell types. Each dot represented a single testicular cell, and it was colored based on the cell types. (B) Expression of the selected markers identifying major testicular cell types on the UMAP plot. Blue or gray represented a higher or lower expression level as shown on the color key at the right bottom. (C) UMAP plot indicated the annotated different types of spermatogonia. Each dot represented a cell with colors according to cell types. The black solid line showed the SSC differentiation trajectory simulated by Monocle 3. Arrow indicated the direction of differentiation. (D) A violin plot illustrated the expression of selected genes used to identify spermatogonial types. (E) Left: Heat map showed the differentially expressed genes (DEGs) for each cluster in (C). The scaled gene expression levels were colored according to *Z* score at upper right. Right: Corresponding top 5 GO (biological process) terms enriched in the DEGs of each spermatogonial cluster. (F) Expression of SSC top 10 DEGs projects to spermatogonial developmental trajectory. APBB1 was found to be enriched in SSCs, and its level was progressively decreased with developmental trajectory. The solid black line was the average expression level of each gene along the developmental trajectory. Each dot represented a cell, and its *Y* value denoted the expression level with colors pursuant to the cell types.

To elucidate the developmental trajectory of human SSCs, we retrieved and analyzed scRNA-seq data from 9 normal adult human testicular tissues, which were obtained from the Gene Expression Omnibus (GEO) datasets (GSE112013, GSE109037, and GSE153947). The messenger RNA (mRNA) profiling of human testicular cells was conducted after filtering and integrating the data using the Seurat program in R. We obtained 25,999 testicular cells and 45,748 genes, which were classified into 14 clusters (Fig. 1A). These clusters were identified by assessing the levels of various testicular cell markers, e.g., *DDX4* for germ cells, *UCHL1* for spermatogonia, *ID4* and *DMRT1* for undifferentiated and differentiating spermatogonia, respectively, *SYCP3* for spermatocytes, *PRM1* for spermatids, *WT1* for Sertoli cells, *MYH11* for peritubular myoid cells, *HSD17B3* for Leydig cells, *VWF* for endothelial cells, and *CD14* for macrophages (Fig. 1B).

To gain insights into the mRNA changes of SSCs during development, we extracted data for spermatogonial clusters and further subdivided them into 7 subgroups using the Seurat R package. We then imported the data into the Monocle 3 R package to construct a developmental trajectory (Fig. 1C). We projected the expression of known markers along with spermatogonia at various phases, including *UCHL1* for spermatogonia, *ID4* for SSCs, *PIWIL4* for early SSCs, and *DMRT1*, *SOHLH2*, and *STRA8* for differentiating spermatogonia. This allowed us to identify 4 SSC clusters (SSC-1 to SSC-4) and 3 Diffing.spg clusters (Diffing.spg-1 to Diffing.spg-3) (Fig. 1D).

We further analyzed the DEGs of spermatogonia at different stages and used Gene Ontology (GO) to analyze the biological processes enriched in each group of cells (Fig. 1E). Notably, we obtained the top 10 DEGs of SSCs onto the developmental trajectory and found that gene *APBB1* was characterized by a distinct expression pattern, since it was only abundantly expressed in the early developmental stages of SSCs and its expression level was gradually decreased along with the differentiation process (Fig. 1F). These results imply that APBB1 might be involved in the fate decisions of SSCs.

### The expression pattern of APBB1 in normal adult testes

**Fig. 2.**
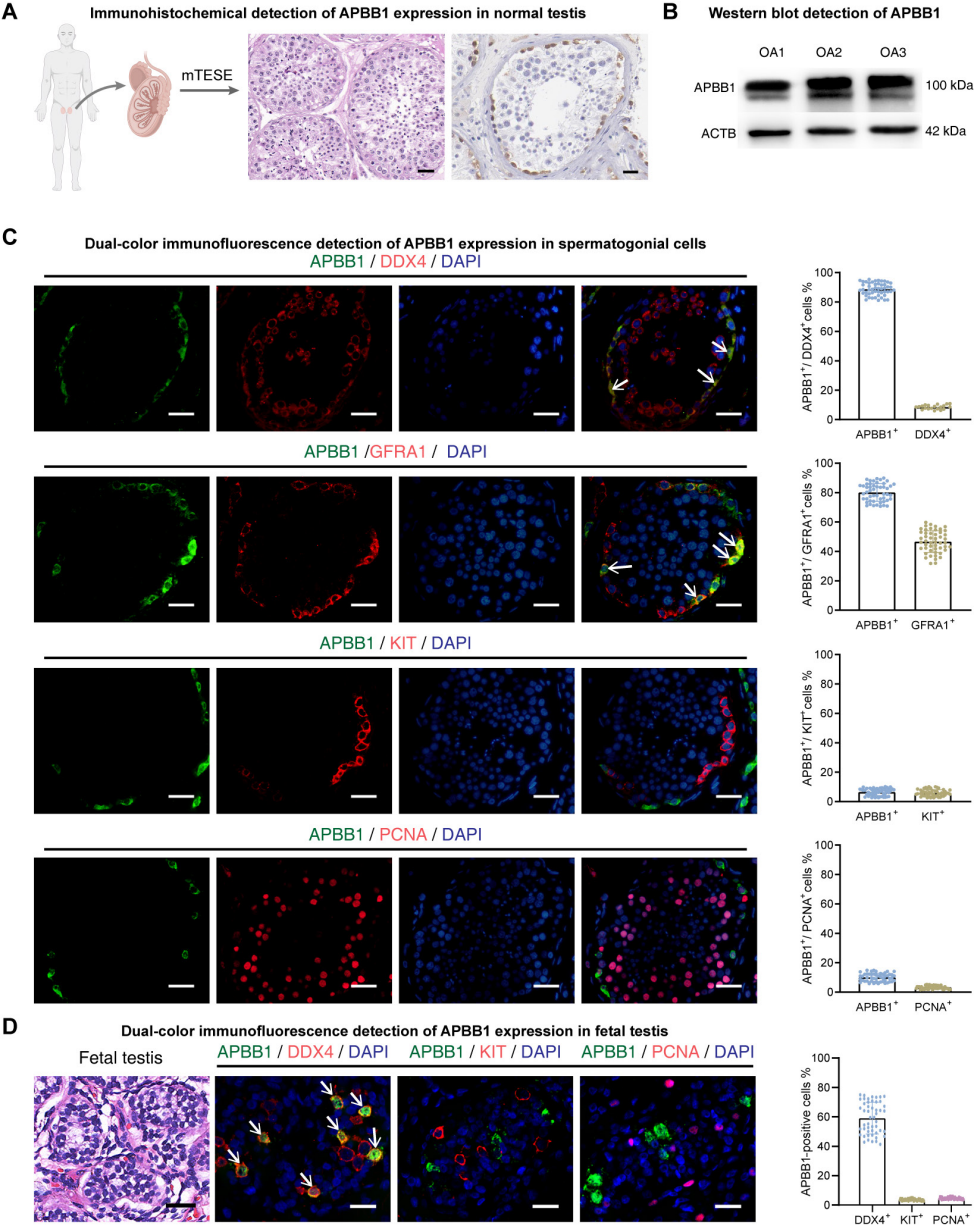
Expression patterns of APBB1 in adult and fetal human testes. (A) H&E staining and APBB1 immunohistochemistry of human testes with normal spermatogenesis. APBB1 was found to be expressed in spermatogonia along the basement membrane of the seminiferous tubules. Scale bars, 50 μm. (B) Western blot detection of APBB1 expression in 3 testes with normal spermatogenesis. (C) Immunofluorescence images of APBB1 with germ cell marker (DDX4), SSC marker (GFRA1), differentiating spermatogonial marker (KIT), and proliferating cell marker (PCNA) in normal human testis. The green signal was APBB1, and the red signals represented different marker molecules, respectively. Cell nuclei were counterstained with DAPI. The bar graphs on the right showed the proportion of APBB1 coexpressed with the marker molecules, respectively. APBB1 was predominantly expressed in quiescent SSCs. White arrows indicated double-positive cells. Scale bars, 50 μm. (D) Left: H&E staining of fetal human testes. Scale bars, 50 μm. Middle: Immunofluorescence images of APBB1 with germ cell marker (DDX4), gonocyte marker (KIT), and proliferating cell marker (PCNA) in fetal testes. The green signal was APBB1, while the red signals represented different marker molecules, respectively. Cellular nuclei were counterstained with DAPI. White arrows denoted double-positive cells. Scale bars, 20 μm. Right: Bar graph demonstrated the coexpression proportion of APBB1 with different markers.

To seek cellular localization of APBB1 in human testes, we obtained testicular tissues from 3 OA patients who underwent micro-testicular sperm extraction (mTESE) surgery. Hematoxylin and eosin (H&E) staining indicated normal spermatogenesis of OA patients (Fig. 2A, middle panel). Immunohistochemistry revealed that APBB1 was primarily present in spermatogonia adjacent to the basement of the seminiferous tubules (Fig. 2A, left panel). Western blot showed the presence of APBB1 in the testes (Fig. 2B). We utilized double immunostaining to observe the cellular localization of APBB1 in human testes with various SSC and spermatogonial markers. Immunohistochemistry demonstrated that 93.15 ± 3.89% (*n* = 50) of APBB1-positive cells were colocalized with DDX4 (a germ cell marker) (Fig. 2C), while 85.58 ± 3.91% (*n* = 50) of APBB1-positive cells coexpressed GFRA1 (an SSC marker) (Fig. 2C). Only 4.93 ± 2.51% (*n* = 50) of APBB1-positive cells were colocalized with KIT (a hallmark for differentiating spermatogonia) (Fig. 2C). Notably, 9.27 ± 3.19% (*n* = 50) of APBB1-positive cells weakly coexpressed PCNA (a cell proliferation marker) (Fig. 2C), which reflects that APBB1 may negatively regulate proliferation of human SSCs (Fig. 2C). Furthermore, we examined the expression of APBB1 in human fetal testes, and we found that APBB1 was predominantly expressed in DDX4^+^ primordial germ cells rather than KIT^+^ gonocytes (Fig. 2D). Interestingly, there was no colocalization of APBB1 with PCNA (Fig. 2D). Together, these findings were consistent with scRNA-seq analysis, suggesting that APBB1 is mainly expressed in human SSCs and may negatively regulate their proliferation.

### The effect of APBB1 on human SSC proliferation and apoptosis

**Fig. 3.**
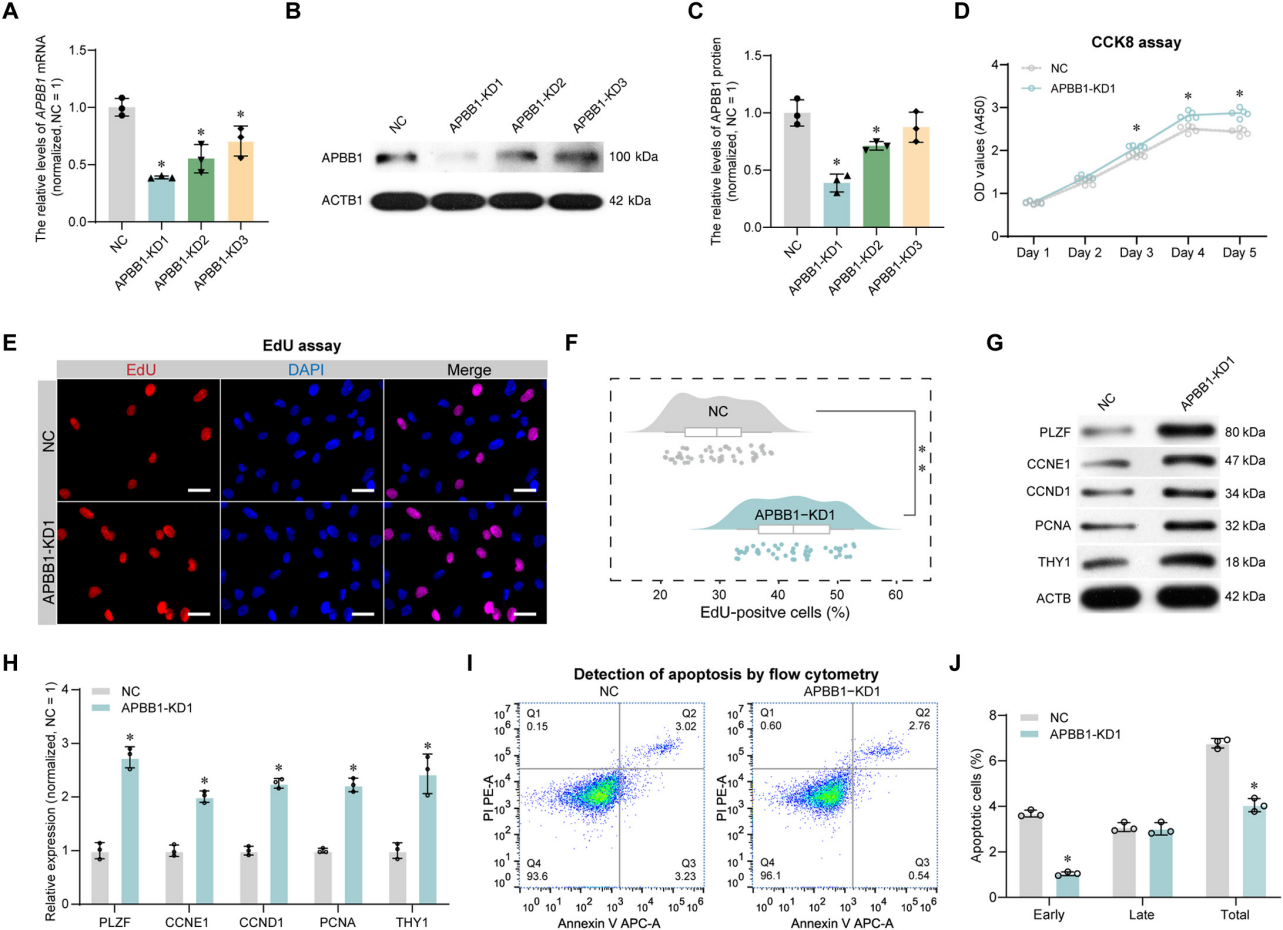
Effect of APBB1 knockdown on proliferation and apoptosis of human SSCs. (A) qPCR detection of *APBB1* mRNA levels in human SSCs transfected with APBB1 shRNAs. Three shRNAs were engineered and interfered with APBB1 expression, and APBB1-KD1 had the best inhibitory effect. (B and C) Western blot assay of APBB1 protein levels in human SSCs transfected with APBB1 shRNAs. The protein levels were lowest in human SSCs by APBB1-KD1 transfection. (D) CCK8 assays were performed to analyze the proliferation ability of human SSCs transfected with APBB1 shRNAs from 1 to 5 d. Cell proliferation was significantly enhanced at days 3 to 5 after APBB1 inhibition. Each small circle represented the data of one detection. (E) EdU incorporation assay was conducted to detect DNA synthesis in human SSCs at 48 h after APBB1 knockdown. Cell nuclei were counterstained with DAPI. (F) Rain cloud plots showed the percentages of EdU-positive cells in (E). Cellular DNA synthesis was significantly increased upon APBB1 knockdown. Each dot represented the result of one picture analysis, and 50 replicates were counted per group. (G) Western blot analysis revealed the decreased expression levels of proteins, including PLZF, CCNE1, CCND1, PCNA, and THY1, in regulating SSC proliferation of human SSCs after APBB1 knockdown. (H) Bar graph indicated the relative levels of each protein in (G) compared to the NC group. (I) Flow cytometry detection of apoptotic cells in human SSCs after APBB1 knockdown. (J) Bar graph displayed the percentages of apoptotic cells in human SSCs after APBB1 knockdown. *P < 0.05; **P < 0.01.

To unveil the roles of APBB1 in regulating the proliferation and apoptosis of human SSCs, we utilized the human SSC line that exhibits biochemical characteristics of primary human SSCs [25,32]. APBB1 silencing in human SSC line was achieved as shown by quantitative polymerase chain reaction (qPCR) (Fig. 3A) and Western blot (Fig. 3B and C) analyses using 3 short hairpin RNAs (shRNAs) targeting *APBB1*, and APBB1-KD1 shRNA exhibited the highest inhibitory effect (APBB1 protein, mean ± SD: 1.00 ± 0.08 versus 0.39 ± 0.02; *P* < 0.05, *t* test). Cellular proliferation was monitored from day 1 to day 5 post-transfection using the Cell Counting Kit-8 (CCK8) assay, and notable enhancement was observed in proliferation of human SSCs by APBB1 knockdown from day 3 to day 5 (*n* = 3; *t* test, *P* < 0.05) (Fig. 3D). The 5-ethynyl-2′-deoxyuridine (EdU) assay was used to evaluate DNA synthesis showing a remarkable increase of human SSCs following APBB1 shRNA1 (KD1) compared to the negative control (NC) group (*n* = 50, mean ± SD: 35.45 ± 2.99% versus 25.48 ± 2.93%; *P* < 0.05, *t* test) (Fig. 3E and F). Additionally, the expression levels of several proteins associated with SSC proliferation and self-renewal, including PLZF, CCNE1, CCND1, PCNA, and THY1, were examined using Western blot analysis. Our results indicated that the APBB1 shRNA1 (KD1) led to an up-regulation of these proteins in human SSCs (*n* = 3; *t* test, *P* < 0.05) (Fig. 3G and H), which implies potential enhancement of self-renewal and proliferation by knockdown. Apoptosis was detected using flow cytometry, which illustrated that APBB1 shRNA1 (KD1) resulted in a notable decrease in early apoptosis (*n* = 3, mean ± SD: 1.04 ± 0.08% versus 3.68 ± 0.15%; *P* < 0.05, *t* test) (Fig. 3I and J). Collectively, these data implicate that APBB1 knockdown enhances the proliferation and suppresses the apoptosis of human SSCs.

### Screening of APBB1 downstream genes

**Fig. 4.**
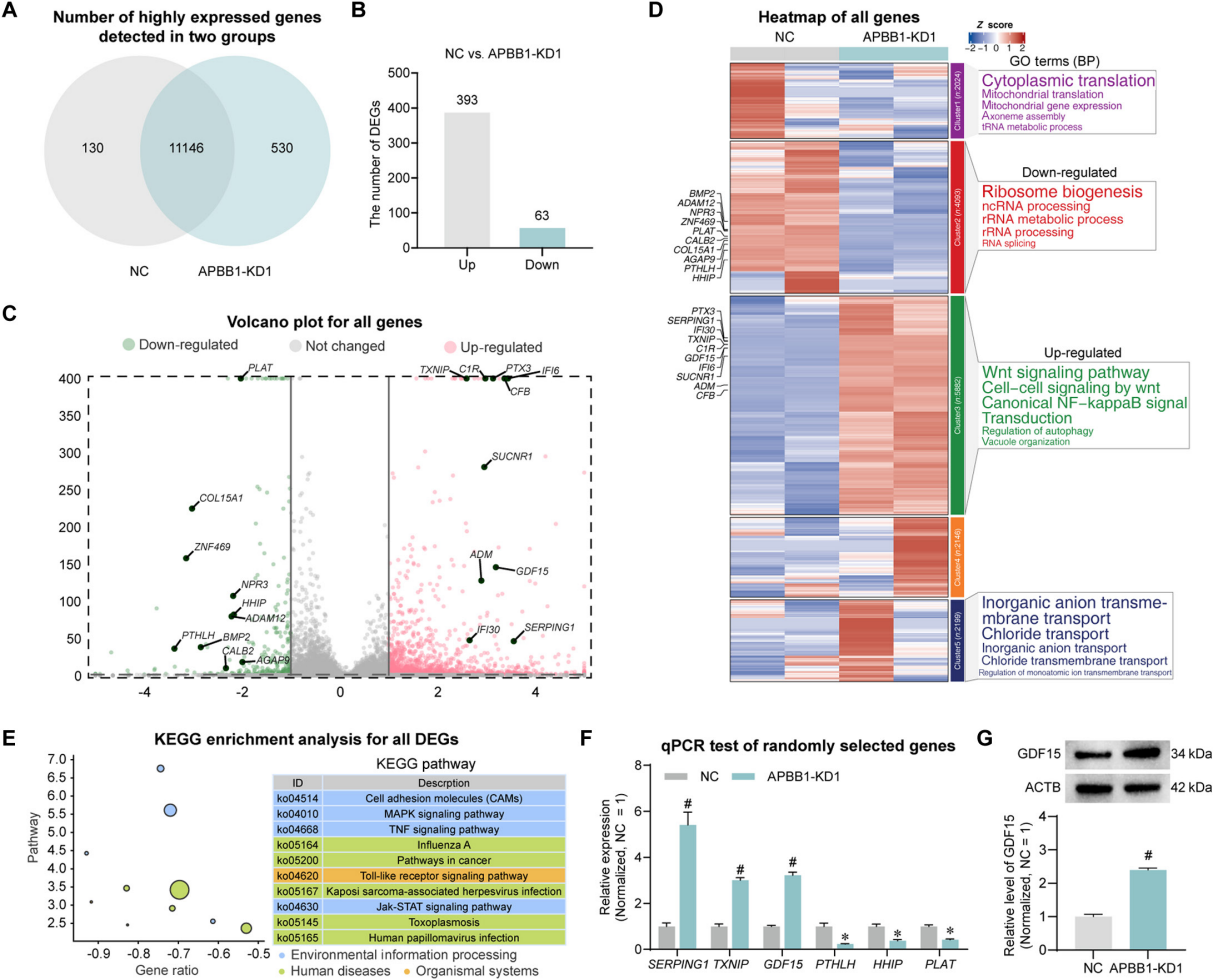
RNA sequencing analysis of downstream genes and enrichment pathways of APBB1 in human SSCs. (A) Wayne diagram showed the number of highly expressed genes identified in human SSCs by the APBB-KD1 and NC groups. (B) Bar graph indicated the number of DEGs in human SSCs between the APBB-KD1 and NC groups. Three hundred ninety-three genes were significantly up-regulated and 63 genes were remarkably down-regulated. (C) Volcano plot illustrated the distribution of all identified genes in (A). Top 20 DEGs were labeled. Green, gray, and red represented the down-regulated, not changed, and up-regulated genes, respectively. (D) Left: Heatmap showed the expression and trends of all genes. The scaled gene expression levels were colored according to *Z* score at upper right. All genes were categorized into 5 clusters, and genes in cluster 2 were significantly down-regulated in human SSCs by APBB1-KD1, where genes in cluster 3 were significantly up-regulated in human SSCs by APBB1-KD1. Top 20 DEGs were labeled. Right: Top 5 GO terms enriched in genes of each cluster. WNT signaling pathway was significantly up-regulated in human SSCs by APBB1 knockdown. (E) All DEGs were analyzed for KEGG enrichment, and top 10 KEGG terms were listed. (F) The qPCR was used to validate the expression of 6 randomly selected DEGs of RNA sequencing. The expression changes of these genes by qPCR were consistent with the RNA sequencing results. (G) Western blot detected the elevated expression of GDF15 protein. *P < 0.05.

To elucidate the molecular mechanisms underlying APBB1's regulation of human SSC proliferation, we conducted RNA sequencing to seek the genes influenced by APBB1. Post-filtering to eliminate genes with very low expression levels, 11,276 genes were seen in the NC group, and 11,676 genes were present in the APBB1-KD1 group (Fig. 4A). The silencing of APBB1 led to a notable up-regulation of 393 genes and down-regulation of 63 genes relative to the NC group (Fig. 4B). A volcano plot was used to visualize the distribution of all genes, and the top 20 DEGs were highlighted and annotated (Fig. 4C). An analysis of gene expression trends generated the categorization of genes into 5 clusters, with cluster 2 genes experiencing significant down-regulation and cluster 3 genes experiencing remarkable up-regulation by APBB1 knockdown. A GO (biological process) analysis across all clusters revealed that APBB1 knockdown significantly reduced the biogenesis of ribosomes, whereas the WNT signaling pathway was markedly enhanced (Fig. 4D). Additionally, a Kyoto Encyclopedia of Genes and Genomes (KEGG) analysis of all DEGs indicated that the knockdown of APBB1 induced substantial alterations in signaling pathways, e.g., the MAPK pathway (Fig. 4E). The qPCR was employed to quantify the expression levels of a subset of randomly chosen DEGs following APBB1 knockdown, such as *GDF15*, *SERPING1*, *TXNIP*, *PTHLH*, *HHIP*, and *PLAT* (*n* = 3; *t* test, *P* < 0.05) (Fig. 4F). The qPCR results were consistent with our RNA sequencing data. Using the STRING database, we conducted a protein–protein interaction (PPI) analysis on the top 50 genes up-regulated by APBB1 knockdown. These genes were categorized into 5 distinct clusters. Based upon differential expression folds, the top 5 hub genes identified were *GDF15*, *C1S*, *CIR*, *SERPING1*, and *IFI6* (Fig. S2A). Subsequent projection of these genes onto the testicular single-cell atlas revealed that *GDF15* was highly expressed in SSCs (Fig. S2B). Western blot showed that APBB1 knockdown resulted in significant up-regulation of GDF15 (*n* = 3; *t* test, *P* < 0.05) (Fig. 4G). Among the genes up-regulated by APBB1 knockdown, GDF15 has been known to participate in both the MAPK and WNT signaling cascades. Considered together, we propose that GDF15 may serve as a downstream target of APBB1.

### GDF15 attenuates phenotypic changes caused by APBB1 knockdown

**Fig. 5.**
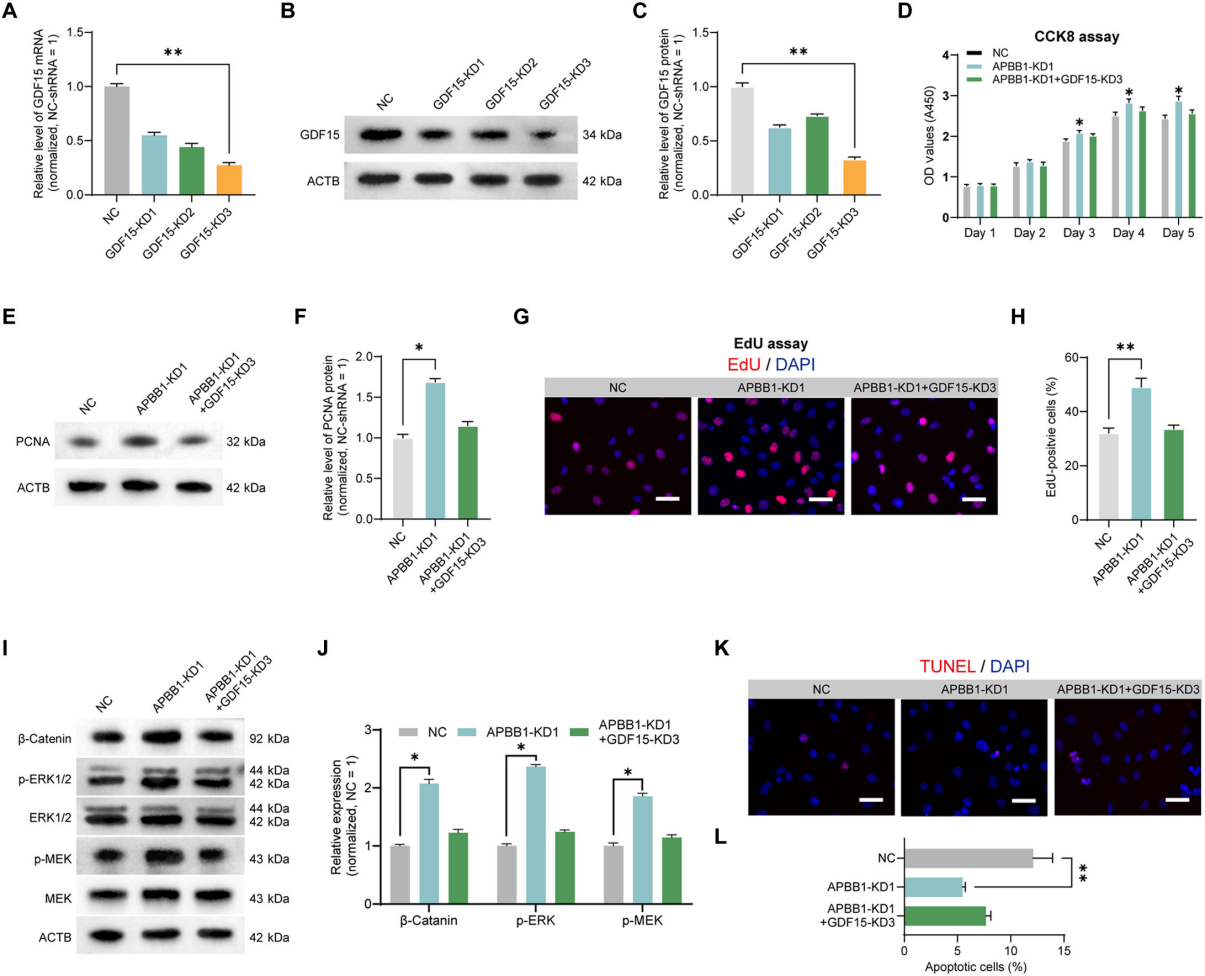
GDF15 alleviated phenotypic changes caused by APBB1 knockdown in human SSCs. (A) The qPCR assay displayed the *GDF15* transcripts in human SSCs by 3 GDF15 shRNAs. The most substantial decrease in *GDF15* mRNA levels was observed in human SSCs after GDF15-KD3 transfection. (B and C) Western blot detection of GDF15 protein levels in human SSCs by 3 GDF15 shRNAs. GDF15-KD3 had the best inhibitory effect on protein levels. (D) CCK8 detected cell proliferation in human SSCs treated with NC, APBB1-KD1, and APBB1-KD1 + GDF15-KD3 from 1 to 5 d. GDF15 inhibition antagonized the increase in SSC proliferation induced by APBB1 knockdown at days 3 to 5 post-transfection. (E and F) Western blot detected the expression level of PCNA proteins in human SSCs treated with NC, APBB1-KD1, and APBB1-KD1 + GDF15-KD3 at 48 h. GDF15 knockdown antagonized the up-regulation of PCNA levels caused by APBB1 inhibition. (G and H) EdU incorporation assay evaluated DNA synthesis in human SSCs treated with NC, APBB1-KD1, and APBB1-KD1 + GDF15-KD3 at 48 h. Cell nuclei were counterstained with DAPI. Scale bars, 20 μm. GDF15 knockdown reversed the increase in DNA synthesis induced by APBB1 inhibition. (I and J) Western blot detection of alterations in WNT and MAPK signaling pathways in human SSCs treated with NC, APBB1-KD1, and APBB1-KD1 + GDF15-KD3. APBB1 knockdown up-regulated β-catenin, a key protein of the WNT pathway, and enhanced the phosphorylation of MEK and ERK1/2, important proteins of the MAPK pathway. Simultaneous knockdown of GDF15 and APBB1 antagonized this change. (K and L) TUNEL (terminal deoxynucleotidyl transferase-mediated deoxyuridine triphosphate nick end labeling) assay detected the apoptosis of human SSCs treated with NC, APBB1-KD1, and APBB1-KD1 + GDF15-KD3. Simultaneous knockdown of GDF15 and APBB1 antagonized this change. Cellular nuclei were counterstained with DAPI. Scale bars, 20 μm. *P < 0.05; **P < 0.01.

**Fig. 6.**
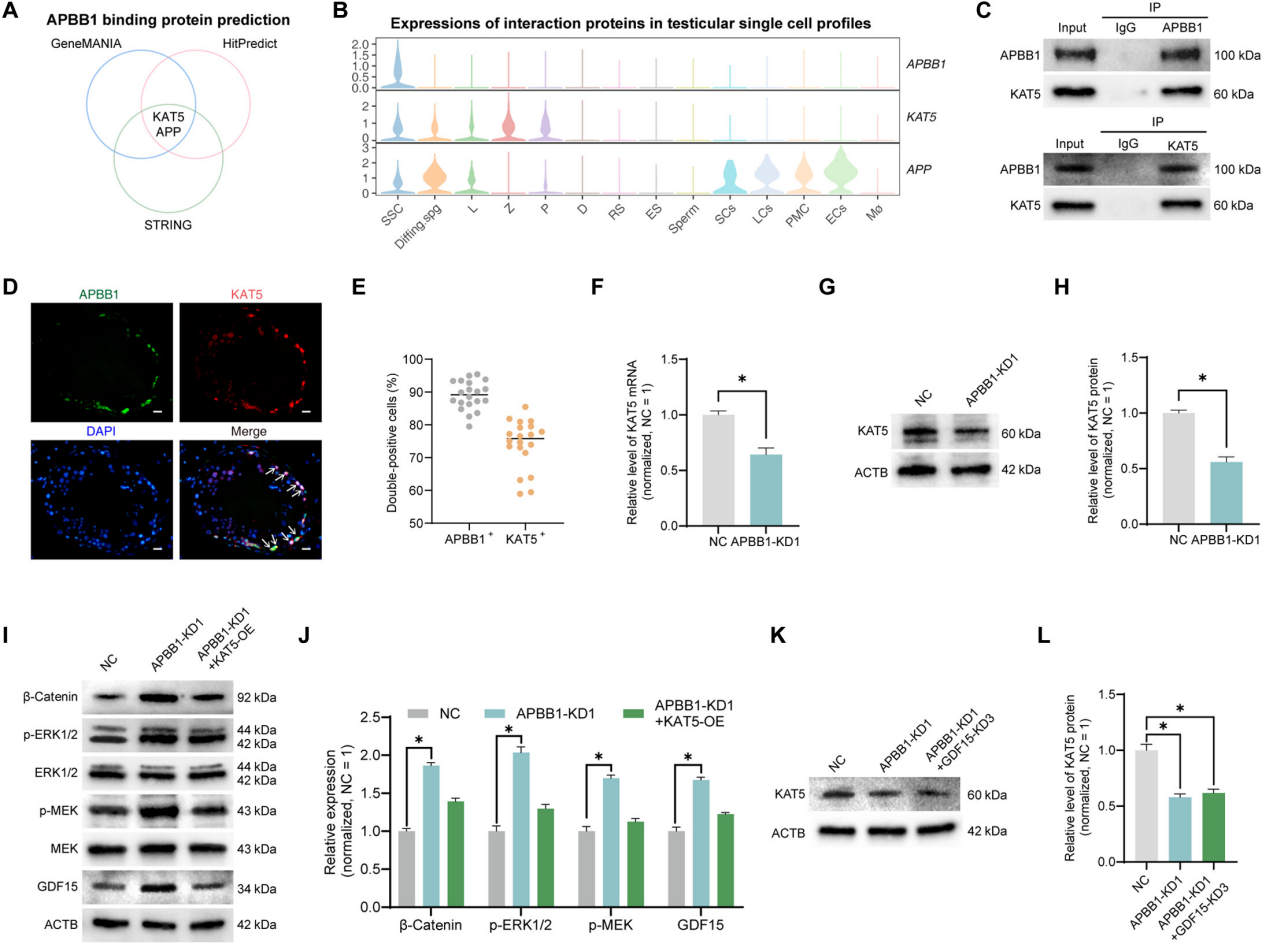
Prediction and validation of APBB1-interacting proteins in human SSCs. (A) Wayne diagram illustrated the intersection of 3 databases on the prediction of APBB1-interacting proteins. All 3 databases predicted that APBB1 interacted with APP and KAT5. (B) Violin plot demonstrated the distribution of APBB1, APP, and KAT in testicular single-cell atlas shown in (A). (C) Co-immunoprecipitation (Co-IP) assay revealed that APBB1 interacted with KAT5 in human SSCs. (D) Immunohistochemistry of APBB1 (green fluorescence) and KAT5 (red fluorescence) in normal human testes. Cellular nuclei were counterstained with DAPI. White arrows indicated double-positive cells. Scale bars, 20 μm. (E) Bar graph demonstrated the percentages of APBB1 coexpressed with KAT5 in normal human testes in (D). APBB1 and KAT5 were colocalized in most SSCs. (F) The qPCR results showed that the mRNA level of *KAT5* was decreased by APBB1 knockdown. (G and H) Western blot revealed KAT5 protein levels in human SSCs after APBB1 knockdown. Bar graphs indicated the relative levels of KAT5 in the APBB1-KD1 human SSCs compared with the NC group. (I and J) Western blot detected the alterations in β-catenin, p-ERK1/2, p-MEK, and GDF15 in human SSCs treated with NC, APBB1-KD1, and APBB1-KD1 + KAT5-OE. APBB1 knockdown increased levels of β-catenin, phosphorylation of MEK and ERK1/2, and GDF15. Simultaneous knockdown of APBB1 and overexpression of KAT5 antagonized this change. (K and L) Western blot implicated protein levels of KAT5 in NC, APBB1-KD1, and APBB1-KD1 + GDF15-KD3 groups. *P < 0.05.

To validate our hypothesis, we employed shRNA to silence GDF15 expression. As demonstrated by qPCR and Western blot analysis, the GDF15-KD3 shRNA exhibited the highest inhibitory effect of *GDF15* mRNA (*n* = 3; *t* test, *P* < 0.01) (Fig. 5A) and protein (Fig. 5B and C) compared to GDF15-KD1-2 shRNAs (*n* = 3; *t* test, *P* < 0.01). We then transfected both APBB1-KD1 and GDF15-KD3 shRNAs to observe phenotypic changes in human SSCs. CCK8 assay showed that APBB1 knockdown significantly increased cell proliferation of human SSCs. However, this increase was blocked in human SSCs by GDF15 knockdown (*n* = 3; *t* test, *P* < 0.05) (Fig. 5D). Similar effect was observed in PCNA expression (*n* = 3; *t* test, *P* < 0.05) (Fig. 5E and F) and EdU incorporation (*n* = 20; *t* test, *P* < 0.05) (Fig. 5G and H) in human SSCs by APBB1 and GDF15 knockdown. RNA sequencing revealed that APBB1 knockdown activated the WNT and MAPK signaling pathways, which are known to promote SSC self-renewal and proliferation [33]. Consequently, we examined the expression levels of key molecules in these pathways. Western blot results indicated that APBB1 knockdown up-regulated β-catenin, a pivotal component of the WNT pathway, as well as the phosphorylation of MAPK kinase (MEK) and ERK1/2 in the MAPK pathway (*n* = 3; *t* test, *P* < 0.05) (Fig. 5I and J). Notably, GDF15 knockdown also counteracted the increased protein levels in human SSCs induced by APBB1 knockdown (*n* = 3; *t* test, *P* < 0.05) (Fig. 6I and J). Furthermore, we detected alterations in apoptosis, and we found that APBB1 knockdown reduced human SSC apoptosis, while GDF15 knockdown diminished the apoptotic effects elicited by APBB1 knockdown (*n* = 3; *t* test, *P* < 0.05) (Fig. 5K and L). Taken together, these findings implicate that GDF15 functions as a downstream of APBB1 and it is involved in the regulation of human SSC fate decisions.

### Prediction and validation of APBB1-interacting proteins

APBB1 has been reported to form complexes with other proteins to regulate gene transcription [34]. To seek the partner of APBB1, we employed GeneMANIA, STRING, and HitPredict databases, and the intersection of the predictions from these platforms indicated potential interactions between APBB1 and KAT5 as well as APP (Fig. 6A). Analysis of scRNA-seq data revealed that both APP and KAT5 were expressed in SSCs, with KAT5 showing higher expression levels in male germ cells compared to somatic cells (Fig. 6B). Protein co-immunoprecipitation (Co-IP) assays were subsequently conducted to assess whether APBB1 indeed binds to APP and KAT5. Our Co-IP assays demonstrated a specific interaction between APBB1 and KAT5 within the human SSCs (Fig. 6C), whereas no binding of APBB1 to APP was observed in these cells (data not shown). Double immunostaining illustrated that nearly 90% of APBB1-expressing cells in human testicular tissues also expressed KAT5 (Fig. 6D and E). Our qPCR and Western blots showed that APBB1 knockdown caused a significant decrease in KAT5 at both mRNA and protein levels (*n* = 3; *t* test, *P* < 0.05) (Fig. 6F to H). Furthermore, we examined the effect of KAT5 on WNT and MAPK signaling pathways. Western blots revealed that KAT5 overexpression notably antagonized the up-regulation of β-catenin, p-ERK1/2, and p-MEK induced by APBB1 knockdown, reflecting that the up-regulation of KAT5 inhibits the activation of WNT and MAPK signaling caused by APBB1 deficiency (*n* = 3; *t* test, *P* < 0.05) (Fig. 6I and J). In addition, we found that KAT5 overexpression suppressed GDF15 up-regulation caused by APBB1 knockdown (*n* = 3; *t* test, *P* < 0.05) (Fig. 6I and J). However, GDF15 down-regulation did not alter the APBB1 knockdown-induced decrease in KAT5 levels (*n* = 3; *t* test, *P* < 0.05) (Fig. 6K and L), suggesting that KAT5 is an upstream molecule of GDF15. These results implicate that APBB1 interacts with KAT5 to regulate GDF15 expression and further modulate WNT and MAPK signaling to affect human SSC proliferation.

### Conditional deletion of *Apbb1* impairs spermatogenesis in mice

To elucidate whether Apbb1 influences spermatogenesis in vivo via the regulation of SSC function, we generated Apbb1 knockout mice. Briefly, LoxP sequences were inserted into the flanking regions of exons 7 to 12 within the mouse *Apbb1* gene and followed by mating with Stra8-GFP-Cre mice to obtain the conditioned Apbb1^−/−^ mice (Fig. S3A). Genotypes were conducted to show *Apbb1* deletion using reverse transcription PCR (RT-PCR) (Fig. S3B), and Apbb1 protein levels were assessed by Western blot and immunofluorescence. Western blot revealed a truncated Apbb1 protein with no expression (Fig. S3C), while immunofluorescence did not detect any signal of Apbb1 (Fig. S3C and D) in the spermatogonia of mouse testes, indicating successful Apbb1 protein knockout.

**Fig. 7.**
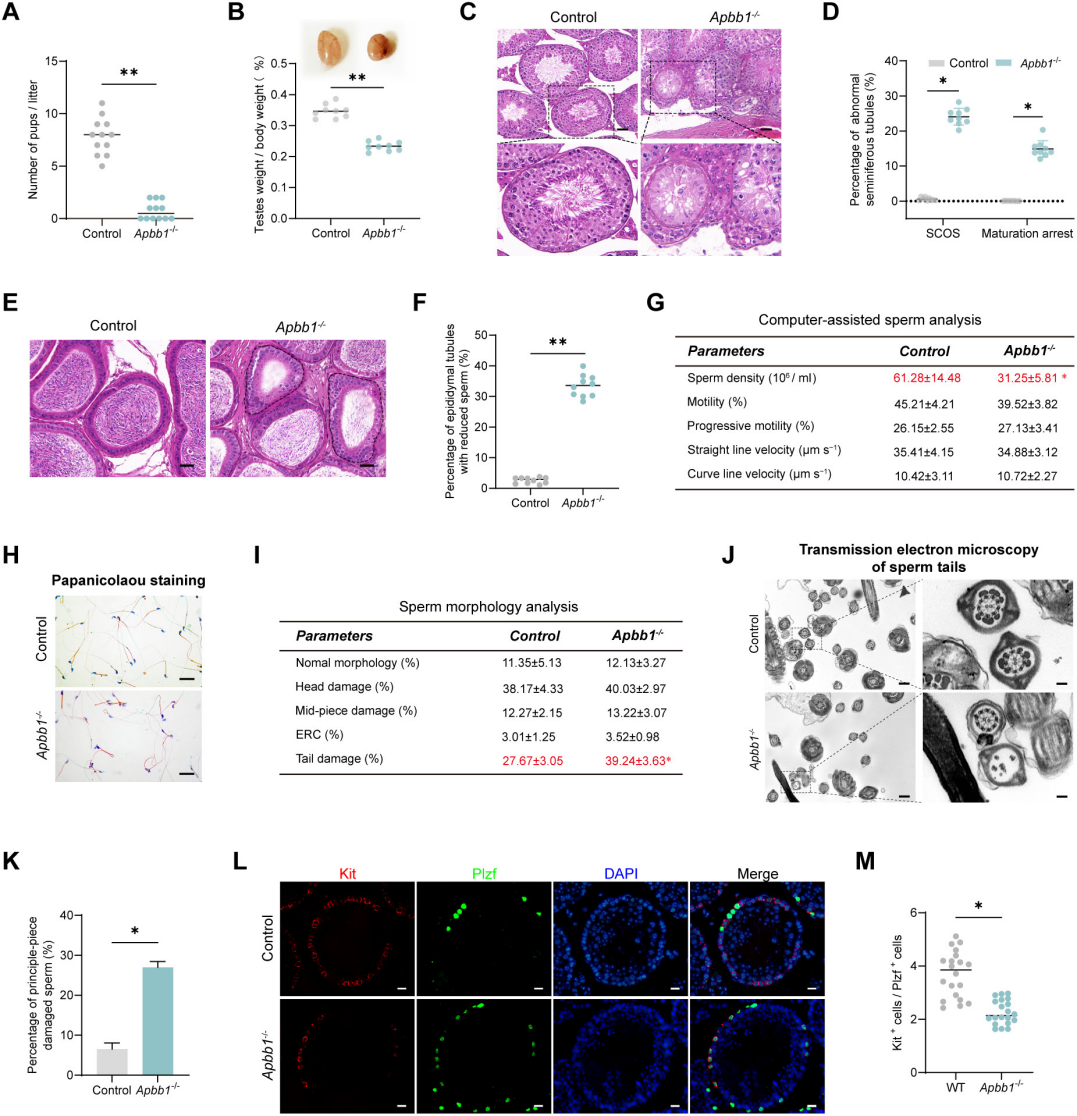
Effect of Apbb1 knockout on fertility and spermatogenesis in mice. (A) Bar graph showed the number of pups per litter in Apbb1^−/−^ and wild-type mice. Apbb1 knockout impaired male fertility in mice. (B) Images of testes from the Apbb1^−/−^ and wild-type mice. The bar graphs indicated the ratio of testes to body weight for each group of mice. Apbb1 knockout caused significant reduction in testicular volume in mice. (C) H&E staining of testes from wild-type and Apbb1^−/−^ mice. The pentagram indicated the seminiferous tubules with abnormal spermatogenesis in Apbb1^−/−^ mice. Scale bars, 50 μm. (D) Dot plot denoted the percentages of different types of abnormal seminiferous tubules. (E) H&E staining of the epididymis of wild-type and Apbb1^−/−^ mice. Apbb1^−/−^ mice had the markedly reduced sperm concentration in many tubules in the tail of the epididymis, with some tubules almost absent of sperm. Pentagrams implied epididymal tubules with abnormal sperm concentration. Scale bars, 50 μm. (F) Dot plot showed the percentages of epididymal ducts with reduced spermatozoa in (E). (G) The table displayed the parameters of sperm concentration and motility in wild-type and Apbb1^−/−^ mice assessed by CASA. Sperm concentration was significantly decreased in Apbb1^−/−^ mice. (H) Images of sperm Papanicolaou staining for morphological assessment of sperm in wild-type and Apbb1^−/−^ mice. Scale bars, 20 μm. (I) The table revealed sperm morphology assessment in wild-type and Apbb1^−/−^ mice in (H). Sperm tail damage was significantly elevated in Apbb1^−/−^ mice. (J) Transmission electron microscopy images of the principal piece of the sperm tail in wild-type and Apbb1^−/−^ mice. Scale bars were 500 nm and 100 nm in the left and right images, respectively. (K) Bar graph showed the proportion of principal piece anomalies in wild-type and Apbb1^−/−^ mice. (L) Immunohistochemistry of Plzf (green) and Kit (red) in wild-type and Apbb1^−/−^ mice. Cellular nuclei were counterstained with DAPI. Scale bars, 20 μm. (M) Dot plot showed the ratio of Kit^+^/Plzf^+^ cells in wild-type and Apbb1^−/−^ mice. This ratio was significantly reduced in Apbb1 knockout mice, reflecting the reduced spermatogonial differentiation. *P < 0.05, ***P < 0.001.

We next determined the impact of Apbb1 deletion on male fertility using the mating experiments with six 8-week-old Apbb1^−/−^ males and control floxed males, housing one male with 2 wild-type females. All control males successfully sired litters (7.83 ± 1.75 offspring per litter, *n* = 12). However, only 3 Apbb1^−/−^ males produced offspring 21 d after copulatory plug detection (1 or 2 offspring per litter), leaving 3 without progeny (*n* = 12; *t* test, *P* < 0.01) (Fig. 7A). The ratio of testis to body weight was significantly lower in Apbb1^−/−^ mice compared to the controls (*n* = 9; *t* test, *P* < 0.01) (Fig. 7B). H&E staining of 16-week-old Apbb1^−/−^ mouse testes revealed an abnormal phenotype of seminiferous tubules, as characterized by a high proportion of Sertoli cells-only symptom (SCOS) (24.25%) and maturation-impaired tubules (16.17%) (*n* = 9; *t* test, *P* < 0.01) (Fig. 7C and D). H&E staining of caudal epididymal sections also showed a significant reduction in sperm concentration by over 30% of tubules (*n* = 10; *t* test, *P* < 0.01) (Fig. 7E and F). Subsequent analysis using computer-assisted semen analysis (CASA) demonstrated that Apbb1^−/−^ mice had significantly reduced sperm concentration and mildly impaired motility (*n* = 5; *t* test, *P* < 0.01) (Fig. 7G). Sperm morphology assays (Papanicolaou staining) indicated that Apbb1 knockout resulted in an increased rate of sperm tail abnormalities, particularly a significant rise in tail curling (*n* = 5; *t* test, *P* < 0.01) (Fig. 7H and I). Transmission electron microscopy further revealed significant impairment of the “9+2” structure in the principal piece of the sperm tails in Apbb1^−/−^ mice (*n* = 3; *t* test, *P* < 0.01) (Fig. 7J and K).

To further characterize alterations in the spermatogonia of Apbb1^−/−^ mice, various antibodies against spermatogonial and Sertoli cell markers were employed for immunostaining, which included Sertoli cells (Sox9^+^), spermatogonia (Uchl1^+^), undifferentiated spermatogonia including SSCs (Plzf^+^), differentiating spermatogonia (Kit^+^), and a proliferation marker (Ki67^+^). Double immunostaining for spermatogonia (Uchl1^+^) and Sertoli cells (Sox9^+^) revealed a significant increase in the ratio of spermatogonia to Sertoli cells in Apbb1^−/−^ mice compared to controls, suggesting that Apbb1 knockout promotes spermatogonial proliferation (*n* = 20, mean ± SD: 0.77 ± 0.15 versus 1.18 ± 0.19; *t* test, *P* < 0.05) (Fig. S4A). Proliferative spermatogonia were further examined by double immunostaining with Uchl1 and Ki67, which showed a significant increase in their proportion (*n* = 20, mean ± SD: 62.64 ± 7.34% versus 84.78 ± 5.06; *t* test, *P* < 0.05) (Fig. S4B). These findings were consistent with our in vitro cell experiments demonstrating that Apbb1 knockdown enhanced spermatogonial proliferation. Notably, double immunostaining of the SSC marker Plzf and differentiating spermatogonial hallmark Kit indicated a significant reduction in the ratio of differentiating to undifferentiated spermatogonia in Apbb1^−/−^ mice compared to controls (*n* = 20, mean ± SD: 3.69 ± 0.87 versus 2.27 ± 0.46; *t* test, *P* < 0.05) (Fig. 7L and M), suggesting that Apbb1 knockout inhibits spermatogonial or SSC differentiation. Collectively, these data suggest that Apbb1 knockout severely impairs mouse fertility, promotes spermatogonial proliferation, and inhibits SSC differentiation.

### Apbb1 knockdown increases SSC self-renewal and inhibits their differentiation in mice

**Fig. 8.**
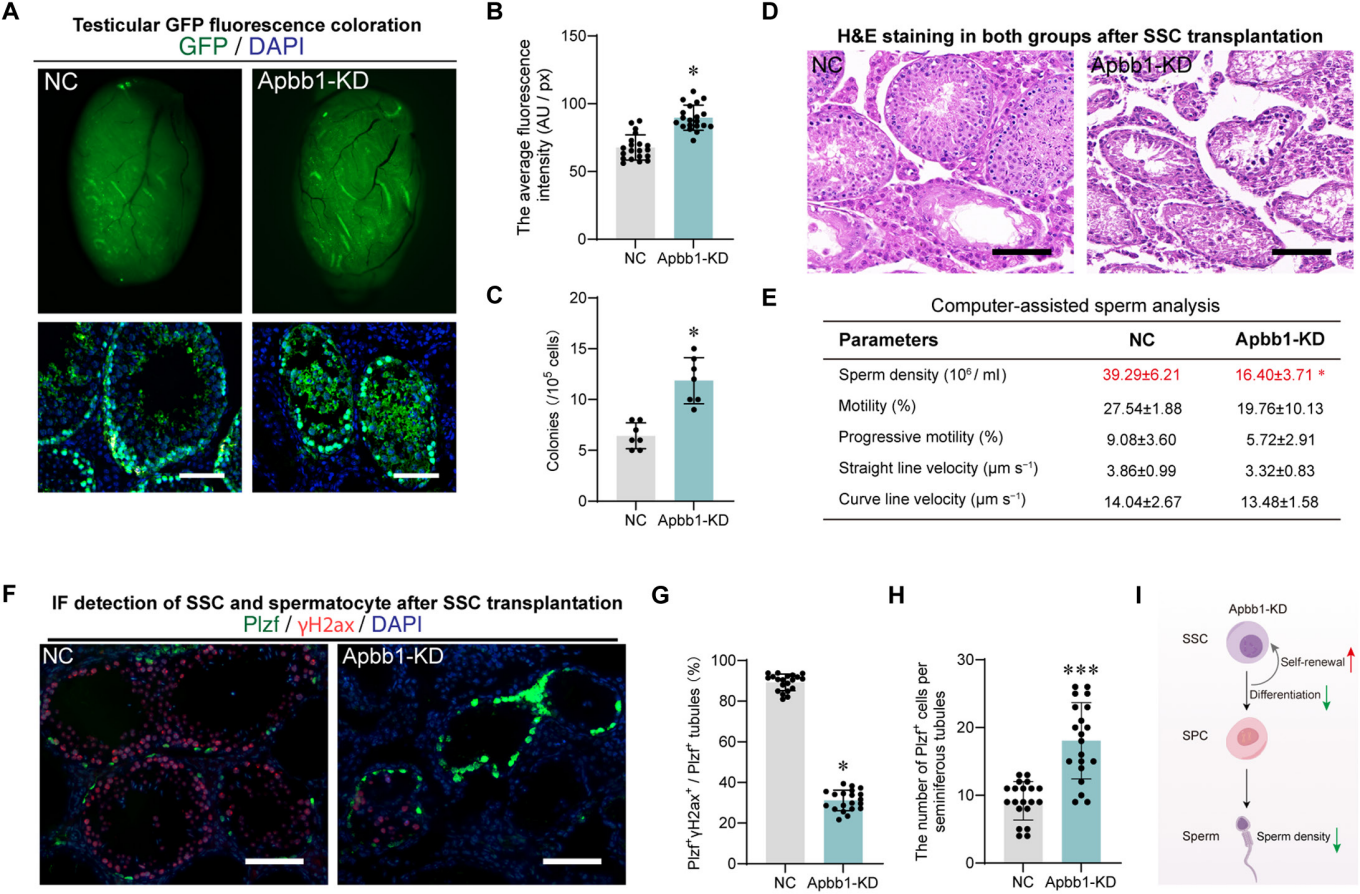
Apbb1 knockdown promoted SSC self-renewal and inhibited differentiation in vivo. (A) Top panel: GFP fluorescence images of testes from recipient mice after transplantation of SSCs with Apbb1 shRNA (Apbb1-KD) and negative control shRNA (NC). Bottom panel: GFP immunofluorescence images of testicular sections from recipient mice after transplantation of SSCs with Apbb1-KD and NC. Scale bars, 100 μm. (B) Bar graph demonstrated the GFP fluorescence intensity of receptor mouse testes calculated by ImageJ in (A). The total testicular GFP fluorescence intensity was higher in the Apbb1-KD group, which implies more SSC colonization. (C) Bar graph illustrated the number of SSC colonies formed after transplantation in (A). (D) H&E staining of testicular sections from recipient mice after transplantation of SSCs with Apbb1-KD and NC. Spermatogenesis reconstruction was inhibited after SSC transplantation in the Apbb1-KD group. (E) Concentration and motility of recipient mouse spermatozoa assessed by CASA after 2 months of SSC transplantation. Sperm concentration was significantly reduced after SSC transplantation in the Apbb1-KD group. (F) Immunofluorescence images of SSC (Plzf^+^) and spermatocytes (γH2ax^+^). Nuclei were counterstained with DAPI. Scale bars, 100 μm. (G) Dot plot showed the ratio of Plzf^+^γH2ax^+^ double-positive tubules to Plzf-positive tubules in (F). This ratio was significantly decreased, suggesting that differentiation of spermatogonia was impaired in the Apbb1-KD group. (H) Dot plot illustrated the mean number of SSCs (Plzf^+^) in seminiferous tubules with Apbb1-KD and NC. The mean number of SSCs was significantly increased, which implicates that self-renewal of SSCs is elevated after Apbb1 knockdown. (I) Schematic diagram illustrated the influence of Apbb1 knockdown on the self-renewal and differentiation of SSCs in vivo. *P < 0.05, ***P < 0.001.

To assess the impact of Apbb1 deficiency on mouse SSC fate decisions in vivo, we conducted transplantation experiments. Mouse SSCs, expressing GFP green fluorescence, were obtained from P. Zhang of Nanjing Medical University. Recipient mice were treated with busulfan to remove endogenous male germ cells. Approximately 90% of the seminiferous tubules in the recipient mice were filled with donor SSCs (5 to 6 μl, 8 × 10^5^ to 10 × 10^5^ cells) via the efferent duct (Fig. 8A). Two months post-transplantation, the testes were collected for spermatogenesis analysis. The overall green fluorescence intensity in the Apbb1 knockdown mice was higher than that in the NC group (*n* = 20, mean ± SD: 67.68 ± 9.34 versus 89.64 ± 9.35; *t* test, *P* < 0.05) (Fig. 8A and B). Apbb1 deficiency enhanced SSC colonization in the recipient testes (*n* = 5, mean ± SD: 6.43 ± 1.27 versus 11.86 ± 2.69; *t* test, *P* < 0.05) (Fig. 8C). H&E staining also indicated that Apbb1 knockdown impaired the ability of SSCs to reconstruct spermatogenesis, with a significantly reduced number of tubules containing intact spermatogenic epithelium compared to the NC group (Fig. 8D). Nevertheless, many seminiferous tubules appeared to have an abundance of SSCs (Fig. 8D). Sperm CASA further revealed a significantly lower sperm concentration in the Apbb1 knockdown group compared to the NC group (*n* = 5; *t* test, *P* < 0.05) (Fig. 8E). To further investigate the phenotypic defects of Apbb1 knockdown tubules, immunostaining was used to detect undifferentiated spermatogonia (Plzf^+^) and spermatocytes (γH2ax^+^). Our results showed a significant reduction in the percentage of γH2ax^+^ spermatocytes in seminiferous tubules (*n* = 20, mean ± SD: 89.19 ± 4.07 versus 31.18 ± 5.04; *t* test, *P* < 0.05) (Fig. 8F and G), while the number of Plzf^+^ undifferentiated spermatogonia was increased significantly in the Apbb1 knockdown group (*n* = 20, mean ± SD: 9.20 ± 2.84 versus 18.05 ± 5.63; *t* test, *P* < 0.001) (Fig. 8F and H). These data suggest that Apbb1 knockdown promotes SSC self-renewal (colonization and proliferation) but inhibits differentiation, ultimately leading to a reduction in spermatozoa production and impairing the ability for initialing spermatogenesis (Fig. 8I).

### Screening of signaling pathways affected by Apbb1 deletion in mouse testes

**Fig. 9.**
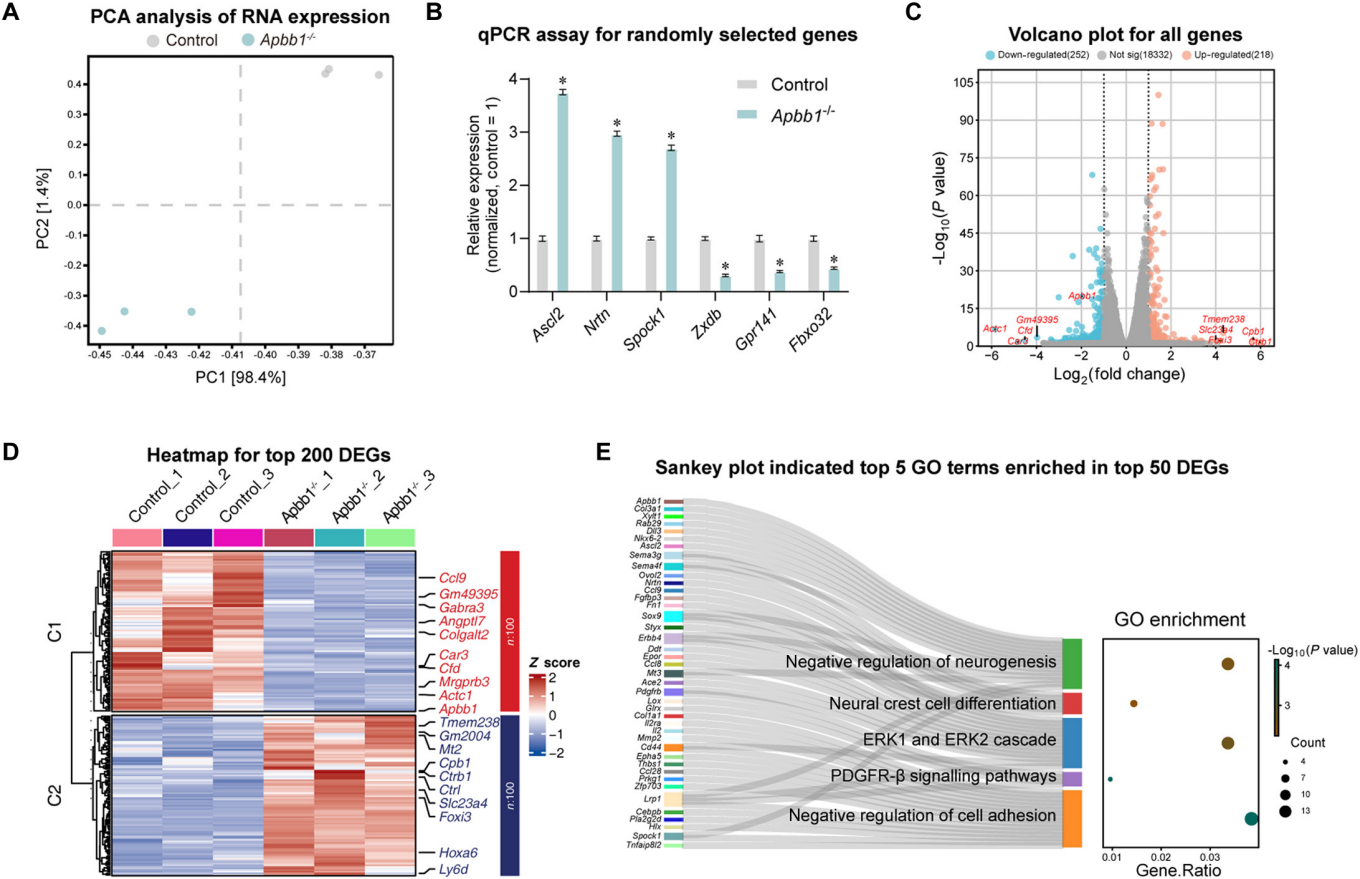
RNA sequencing analyzed transcriptomic changes in Apbb1^−/−^ testes. (A) Dot plot showed principal components analysis (PCA) of top 1,000 genes in Apbb1^−/−^ and control mice. Each point represented a sample. (B) The qPCR verified the expression level of randomly selected DEGs. (C) Volcano plot illustrated distribution of all genes. The genes that were significantly down-regulated were shown in blue, whereas those that were significantly up-regulated were indicated in orange. Top 10 DEGs were labeled. (D) Heatmap displayed the expression levels of top 200 DEGs in wild-type and Apbb1^−/−^ mice. Top 20 DEGs were labeled. The scaled gene expression levels were colored according to *Z* score at right. (E) Sankey plot indicated top 5 GO terms enriched in top 50 DEGs. Left: Top 50 DEGs in wild-type and Apbb1^−/−^ mice. Middle: Top 5 GO terms enriched in these DEGs. Each term was connected to the contained genes through a channel. Right: Each circle represented a GO term, colored by −log_10_ (*P* value), and sized pursuant to the number of gene counts.

To find the signal pathways that are involved in abnormal spermatogenesis in vivo due to Apbb1 deletion, RNA sequencing was conducted with Apbb1^−/−^ testes. After filtering out lowly expressed genes, 19,796 genes were identified. Principal components analysis (PCA) of the top 1,000 genes from both the control and Apbb1^−/−^ mouse testes displayed the substantial transcriptomic difference between these 2 groups (Fig. 9A). The qPCR was used to randomly verify the expression levels of several genes, including *Ascl2*, *Nrtn*, *Spock1*, *Zxdb*, *Gpr141*, and *Fbxo32* (*n* = 3; *t* test, *P* < 0.05) (Fig. 9B)*,* which were consistent with the RNA sequencing results, confirming their reliability. The expression distribution of all genes was illustrated in a volcano plot, where Apbb1 knockdown led to the up-regulation of 218 genes (represented in red) and down-regulation of 252 genes (indicated in blue) (Fig. 9C). Differential gene expression across each sample was depicted by a heatmap (Fig. 9D). Additionally, GO analysis was performed on all DEGs, revealing that Apbb1 knockdown affected the pathways related to differentiation, e.g., negative regulation of gametogenesis and neural crest cell differentiation (Fig. 9E), which was in agreement with our findings from animal models and transplantation experiments. Furthermore, Apbb1 knockdown influenced the ERK1 and ERK2 pathways (Fig. 9E), which was consistent with our RNA sequencing results from human SSCs. Together, these results suggest that Apbb1 knockdown may disrupt the fate decisions of SSCs and spermatogenesis through ERK1/2 signaling pathway.

### Mutation screening and expression levels of the APBB1 in patients with NOA

**Fig. 10.**
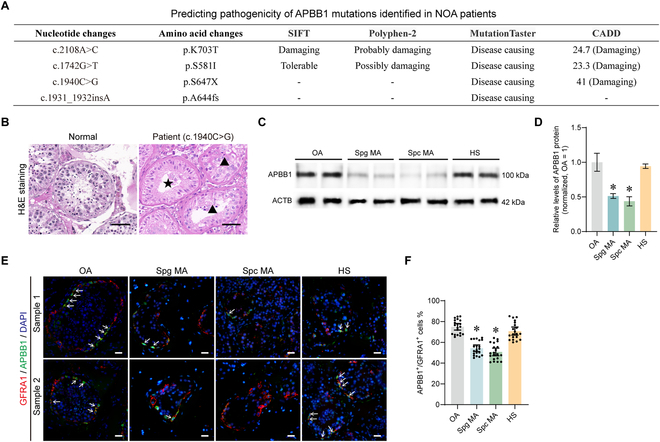
Expression of APBB1 in patients with OA and NOA. (A) Prediction of pathogenicity for *APBB1* mutation sites. Four different softwares were used to evaluate each mutation site and summarized the results in a table. Four potential APBB1 pathogenic mutation sites were detected. (B) H&E staining of testes from OA (normal spermatogenesis) and NOA patients (c.1940C>G), pentagram indicated tubules with male germ cell loss, and triangles denoted tubules with impaired germ cell maturation. Scale bar, 50 μm. (C) Western blot was performed to detect APBB1 expression in testes from OA and NOA patients, including maturation arrest at spermatogonia (Spg MA), maturation arrest at spermatocytes (Spc MA), and hypospermatogenesis (HS). (D) Bar graphs demonstrated the relative levels of APBB1 protein in testes from OA and NOA patients, including Spg MA, Spc MA, and HS. The expression level of APBB1 was significantly down-regulated in Spg MA and Spc MA. (E) Immunofluorescence images of APBB1 and GFRA1 (SSC marker) in seminiferous tubules of testes from OA and NOA patients, including Spg MA, Spc MA, and HS. White arrows indicated double-positive SSCs. Scale bars, 20 μm. (F) Bar plot showed the positive rate of APBB1 in SSCs of seminiferous tubules from OA and NOA patients, including Spg MA, Spc MA, and HS. The percentage of APBB1-positive SSC was significantly reduced in NOA patients with Spg MA or Spc MA. Each dot represented a counting result. *P < 0.05.

To evaluate the potential importance of APBB1 in male fertility, we conducted a mutation screening using whole-exome sequencing (WES) data from NOA patients. The detailed results were presented in Fig. 10A and Tables S4 and S5. In 2,047 NOA patients, we identified 9 individuals with 8 heterozygous variants of *APBB1* (Table S4). The pathogenicity of these APBB1 mutations was predicted using bioinformatics tools, e.g., SIFT and CADD, revealing 4 notable mutations, namely, c.2108A>C, c.1742G>T, c.1940C>G, and c.1931_1932insA (Fig. 10A). Notably, the testicular pathology observed in NOA patients with the c.1940C>G mutation resembled that of Apbb1^−/−^ mice, as characterized by male germ cell loss (labeled with pentagram) or impaired maturation of spermatogenic cells (labeled with triangles) in some seminiferous tubules (Fig. 10B). Furthermore, testicular tissues were obtained from patients diagnosed with NOA who underwent micro-sperm retrieval surgery. These tissues were subsequently categorized based on the outcomes of H&E staining and Johnsen scores encompassing categories, including OA (normal spermatogenesis), maturation at spermatogonia (Spg MA), maturation at spermatocytes (Spc MA), and hypospermatogenesis (HS) (Fig. S5). Western blot analysis showed that APBB1 expression level was notably diminished in seminiferous tubules exhibiting abnormal spermatogenesis, with a particularly pronounced down-regulation in Spg MA and Spc MA testes (*n* = 3; *t* test, *P* < 0.05) (Fig. 10C and D), which was similar to the phenotype of Apbb1^−/−^ mice. Furthermore, immunofluorescence of the distribution of APBB1 in SSCs (GFRA1^+^) revealed a substantially decreased positive rate of APBB1 in SSCs of seminiferous tubules with Spg MA and Spc MA (*n* = 20; *t* test, *P* < 0.05) (Fig. 10E and F). Collectively, these findings imply that APBB1 may be implicated in abnormalities associated with human spermatogenesis, particularly those related to germ cell differentiation disorders.

## Discussion

Within the testis, SSCs maintain the balance between self-renewal and differentiation to maintain the stem cell pool and persistently generate progenitor cells necessary for normal spermatogenesis [35]. Numerous molecules regulating the fate decisions of SSCs have been identified in mice using in vitro culture and transplantation techniques [36]. However, molecular mechanisms governing human SSC self-renewal and differentiation remain largely elusive. Human primary SSCs exhibit a diminished proliferative capacity during culture, and they are difficult to be expanded in vitro [37]. Furthermore, limited sources of human testicular tissues impede comprehensive studies of human SSCs. To elucidate the regulatory mechanisms underlying fate determinations of human SSCs, we analyzed scRNA-seq data and discovered that APBB1 is selectively expressed in human SSCs, which is consistent with the data by our immunohistological staining. We further elucidated the roles of APBB1 in regulation of SSCs and spermatogenesis through cellular and molecular assays and APBB1 knockout mouse models. Interestingly, we found that APBB1 was associated with KAT5 and inhibited human SSC proliferation by modulating GDF15. In mice, Apbb1 deletion led to an enhancement in self-renewal of SSCs and reduction in their differentiation, which obviously decreased fertility.

APBB1, also known as FE65, was initially identified as a molecule integral to brain development [26]. It can activate or repress gene transcription independently or in conjunction with other molecules, e.g., KAT5, Notch intracellular domain (NICD), and the APP intracellular domain (AICD) [34]. The AICD-APBB1 complex, in conjunction with KAT5, constitutes a transcriptional regulatory module. This complex has been demonstrated to modulate the expression of genes critical for neurogenesis, e.g., *Stathmin* [26]. Our findings suggest that APBB1 has an interaction with KAT5, potentially binding to KAT5 and functioning independently in SSCs.

Upon APBB1 knockdown in human SSCs, we observed a notable increase in cell proliferation. Similarly, in the testes of Apbb1 knockout mice, we revealed an abnormal surge in SSC self-renewal (proliferation and colonization). The total number of SSCs was significantly elevated in Apbb1^−/−^ mice, as evidenced by our observations that the ratio of Plzf-positive SSCs to Kit-positive differentiating spermatogonia was markedly increased by Apbb1 knockout. It has been reported that AICD/APBB1 double transgenic mice exhibit a reduced number of immature neurons and the decrease in the proliferation of hippocampal progenitor cells [38]. This study indicates that Apbb1 overexpression conversely reduces the number of undifferentiated neurons [38], which is consistent with our findings that Apbb1 negatively regulates the self-renewal of human SSCs.

After knocking out the *Apbb1* gene in mouse testes, the maintenance of the SSC pool is disrupted. SSCs became more self-renewing to hinder their differentiation, leading to spermatogenesis disorder. Most seminiferous tubules showed loss of male germ cells, while few spermatids were observed in other tubules. This finding suggests that the function of Apbb1 in regulating spermatogenesis may be partially compensated by other signaling pathways. Apbb1 belongs to the Apbb protein family, which includes 3 members, namely, Apbb1, Apbb2, and Apbb3 [26]. These 3 members share a conserved structure featuring a tryptophan–tryptophan (WW) domain and 2 successive phosphor-tyrosine-binding (PTB) domains [39]. The scRNA-seq analysis indicates that all 3 genes are expressed in the human testis, with APBB1 primarily expressed in SSCs, APBB2 mainly in peritubular myoid-like cells, and APBB3 predominantly in spermatocytes. Notably, APBB2 and APBB3 were also expressed in spermatogonia at low levels (data not shown). It has been reported that all 3 APBB proteins can bind to AICD through the PTB2 domain and to KAT5 through the PTB1 domain [40]. Knockout of either *Apbb1* or *Apbb2* alone in mice does not significantly affect neural development; however, simultaneous knockout of both genes leads to significant neural developmental defect [41]. These results implicate that there is functional compensation among the Apbb protein family, which may explain why Apbb1^−/−^ mice still have certain spermatogenesis. Future studies would further elucidate the specific roles of APBB proteins and their interaction in controlling spermatogenesis and human SSC development.

Using human SSC line, we observed that APBB1 knockdown promoted cell proliferation. RNA sequencing revealed that APBB1 knockdown activated WNT and ERK1/2 signaling pathways, which is essential for SSC self-renewal [33]. Among the up-regulated genes by APBB1 knockdown, *GDF15* has been shown to stimulate cell growth and it is involved in the regulatory processes of WNT [42] and MAPK signaling [43]. Although we have demonstrated an important role of GDF15 in APBB1-mediated SSC self-renewal and apoptosis, there are other hub genes that may participate in the PPI network of up-regulated genes. For instance, EPAS1, also known as HIF-2α, is involved in the regulation of hypoxia-inducible factor (HIF) signaling, and it is essential for SSC regeneration conditions [44]. The ubiquitin-like protein ISG15 has been thought to be a central player in the host antiviral response [45], and it is highly expressed in cancer stem cells and promotes tumor growth [46]; however, whether ISG15 controls SSC proliferation remains to be investigated. Additionally, we observed that PTN [47] and EGR1 [48] were up-regulated by APBB1 knockdown.

Through protein interaction prediction and Co-IP experiments, we have identified that APBB1 interacted with KAT5 and they participated in regulation of human SSCs. Nevertheless, it remains to be explored systematically and unbiasedly the molecular mechanisms of APBB1 in regulating the fate decisions of human SSCs. In addition to the interaction with KAT5, it has been suggested that APBB1 can bind to APP, APLP1, CP2, LSF, LBP1, and SET, which contributes to transcriptional processes [40]. Moreover, APBB1 has been reported to form complexes with Mena and Evl, participating in cytoskeletal regulation and cell migration activities [49]. Despite these findings, further investigation is required to determine whether APBB1 influences human SSC development through the aforementioned or unknown mechanisms. For instance, immunoprecipitation mass spectrometry (IP-MS) may be employed to conduct a thorough screening of potential molecular partners of APBB1, which could provide deeper insights into the functions and mechanisms of APBB1 in controlling the fate determinations of human SSCs.

Notably, Apbb1 knockout resulted in remarkably reduced fertility in mice. The pathological features in the testes were characterized by impaired germ cell maturation or germ cell loss in part of the seminiferous tubules, resembling the pathological features of human azoospermia. Our findings indicate that overall expression levels and positivity of Apbb1 in testicular tissues from patients with NOA were significantly down-regulated. We have also conducted genetic testing on 2,047 patients with NOA and have identified 4 meaningful *APBB1* mutations. In particular, the testicular pathologic features of NOA patients with the C.2108A>C mutation were similar to those of Apbb1^−/−^ mice, which implicates that the C.2108A>C mutation may lead to spermatogenesis disorder. It would be interesting to further determinate whether *APBB1* mutations lead to abnormal development of human SSCs or spermatogenesis failure.

In summary, we have delineated for the first time the specific expression of APBB1 in human SSCs. We have further demonstrated that APBB1 in concert with KAT5 forms a complex that suppresses GDF15 expression, thereby modulating the WNT and MAPK signaling pathways to negatively control SSC self-renewal, which is essential for retaining the homeostasis of the SSC pool. Notably, the deletion of Apbb1 in mice results in profound spermatogenic failure and male infertility. Furthermore, we identified several pathogenic mutations of *APBB1* in patients with NOA and a down-regulation of APBB1 expression level in these patients, highlighting an association of APBB1 abnormality with NOA. As such, this study offers substantial insights into the complex regulatory mechanisms of SSC fate decisions and spermatogenesis and it may provide a scientific basis for gene therapy of male infertility.

## Materials and Methods

### Experimental design and procedures

This study design comprised 4 distinct sections, as we illustrated in Fig. S1. Employing scRNA-seq analysis and immunohistochemistry, we observed differential expression of APBB1 in human SSCs. Through a combination of in vitro cell culture experiments, mouse SSC transplantation, and Apbb1 knockout mouse model, we elucidated the function of APBB1 in both SSC development and spermatogenesis. Utilizing Co-IP and RNA sequencing, we discovered that APBB1 interacted with KAT5 to modulate GDF15 expression. In NOA patients, APBB1 was markedly down-regulated, and its association with pathogenic mutations was detected, suggesting a potential link between APBB1 dysregulation and NOA.

### Ethics statement and human testicular tissues’ collection

Human testicular tissues from OA and NOA patients utilized in this study were approved by the Ethics Committee of the School of Basic Medical Science at Central South University (no. 2022-KT25). All participants provided their informed consent through a signed statement. Human testicular samples were obtained from 18 NOA patients at 28 to 48 years old undergoing mTESE surgery. The animal experimental study was authorized by the Experimental Animal Welfare Ethics Committee of Central South University (XMSB-2022-0053) in terms of the principles of animal protection, welfare, ethics, and the relevant national guidelines on experimental animal welfare ethics.

### Acquisition and analysis of scRNA-seq data of human testicular tissues

The normal testicular cell scRNA-seq datasets (GSE112013 [50], GSE109037 [51], and GSE153947 [52]) were obtained from the GEO dataset repository (https://www.ncbi.nlm.nih.gov/gds). The Seurat 4.4 R package was utilized for the analysis of these scRNA-seq data [53]. Initially, the expression matrix data were imported using the Read10X and read.table functions, and Seurat objects were created for each matrix. Subsequently, the data within these objects were filtered and normalized. Cells with gene numbers between 500 and 8,000 and with a proportion of mitochondrial genes less than 20% were retained, and ribosomal genes were manually eliminated. The Find Integration Anchors function was utilized to remove batch effect between different datasets, and 2,000 characterized genes were acquired. Following this process, the IntegrateData function was applied to merge the data. Subsequent clustering parameters were set to the default values of the seurat R package. The integrated Seurat object was subjected to downscaling and clustering analysis utilizing the uniform manifold approximation and projection (UMAP), and each cluster was identified by marker genes. Furthermore, developmental trajectory analysis of SSCs was performed using Monocle 3 (https://cole-trapnell-lab.github.io/monocle3/). The ClusterGvis R package (https://github.com/junjunlab/ClusterGVis) was employed for the generation of heatmaps and conducting GO analyses. All the point and line plots were optimized using ggplot2 (https://www.rdocumentation.org/packages/ggplot2, version 3.4.0).

### Source and culture of human SSCs

The human SSC line was established previously by our research team [32]. The culture medium for human SSC line consisted of Dulbecco’s modified Eagle’s medium (DMEM)/F12 (Gibco, Grand Island, NY, USA) supplemented with 10% fetal bovine serum (FBS; Gibco), while the addition of antibiotics was optional. The human SSC line was subcultured every 2 to 3 d at 34 °C and 5% CO_2_.

### RNA extraction and qPCR

RNA was harvested from the cells with the RNAiso Plus reagent (Takara, Tokyo, Japan). The purity and quantity of the isolated RNA were determined using a NanoDrop spectrophotometer (Thermo Fisher Scientific). Subsequently, complementary DNA (cDNA) was synthesized using commercial kits (Roche, Basel, Switzerland) for RT. The qPCR was conducted following the manufacturer’s protocol on the ABI Prism 7700 system (Applied Biosystems). To quantify the relative mRNA levels, the 2^−ΔΔ(Ct)^ method was employed with *beta-actin* (*ACTB*) serving as an internal reference. After comprehensive analysis of each sample, 3 replicates were conducted, and the average results were calculated. The primers of genes were sourced from PrimerBank (https://pga.mgh.harvard.edu/primerbank), and their sequences were listed in Table S1.

### Immunohistochemistry

For immunohistochemistry, testis sections were deparaffinized using xylene and rehydrated with a series of graded ethanol solutions. Subsequently, the sections were heated for 18 min at 98 °C in 0.01 M sodium citrate buffer. Endogenous peroxidase activities were blocked by 3% hydrogen peroxide (Zsbio, Beijing, China). The tissues were then incubated with 0.25% Triton X-100 for 15 min at room temperature to increase tissue permeability. Afterward, tissues were blocked for 1 h at room temperature with 5% bovine serum albumin (BSA). Tissue sections were incubated with the primary antibodies (Table S2) for at least 16 h at 4 °C. Following 3 washes with phosphate-buffered saline (PBS), tissue sections were incubated with a secondary antibody for 1 h at room temperature. The Diaminobenzidine (DAB) kit (Zsbio, Beijing, China) was used to develop the immunostaining. For immunofluorescence, Alexa Fluor-conjugated secondary antibodies were incubated at room temperature for 1 h and followed by nuclear counterstaining with 4′,6-diamidino-2-phenylindole (DAPI). At the end of the process, image capture and analysis of tissue sections were performed using a Zeiss microscope (Carl Zeiss, Germany).

### Transfection of plasmids and shRNAs

The shRNAs were designed and synthesized by Zorin (Shanghai, China) and RiboBio (Guangzhou, China). In accordance with the manufacture’s manual, human SSCs were transfected with shRNA and plasmids at a concentration of 3 μg/ml by Lipofectamine 3000 (Life Technologies). Gene and protein expression levels were assessed at 48 h post-transfection.

### CCK8 assay

The proliferation of human SSCs was assessed by the CCK8 according to the manufacturer’s protocol (Dojindo, Kumamoto, Japan). Specifically, 10% CCK8 reagent was added into the culture medium of human SSCs and incubated for 3 h. Subsequently, the absorbance at 450 nm was measured using a microplate reader (Thermo Fisher Scientific).

### The EdU incorporation assay

Human SSCs were cultured with DMEM/F12 without FBS, and 50 μM EdU (RiboBio) reagent was added into the cell culture medium and incubated for 12 h. Cells were then rinsed with DMEM and fixed by 4% paraformaldehyde. Subsequently, after neutralization with glycine (2 mg/ml), the cells were permeabilized with 0.5% Triton X-100 for 10 min at room temperature. Color development was facilitated using Apollo, while cell nuclei were counterstained with DAPI. Image capture and analysis were conducted utilizing fluorescence microscopy (Zeiss). A minimum of 500 cells were evaluated for statistical analysis.

### Flow cytometry for apoptosis detection

After 48 h of shRNA transfection, human SSCs were collected and washed twice with ice-cold PBS. Following centrifugation, at least 1 million cells were resuspended in Annexin V binding buffer (BD Biosciences, San Jose, CA, USA) according to the manufacturer’s instructions. The cells were then incubated with 10 μl of propidium iodide (PI) and 5 μl of allophycocyanin-labeled Annexin V reagent for 15 min at room temperature to prevent from light. The cells’ apoptosis was subsequently analyzed using a C6 flow cytometer (BD Biosciences).

### Terminal deoxynucleotidyl transferase-mediated deoxyuridine triphosphate nick end labeling assay

After 48 h of shRNA transfection, the cells were assayed for DNA breaks and cell apoptosis using an in situ cell death detection kit (Roche, Mannheim, Germany). Briefly, human SSCs were incubated at room temperature for 15 min with proteinase K (20 mg/ml). Following 3 washes with PBS, the cells were incubated with deoxyuridine triphosphate labeling/terminal deoxynucleotidyl transferase enzyme buffer for 1 h to protect from light. DAPI was used to counterstain cell nuclei. Image capture and analysis were conducted using a fluorescence microscope (Zeiss). At least 500 cells were counted for statistical analysis.

### Western blots and IP assays

Cells and testicular tissues were homogenized and lysed with radioimmunoprecipitation assay (RIPA) lysis buffer (Thermo Fisher Scientific) for 30 min on ice. Subsequently, they were centrifuged at 12,000*g* to obtain clear lysates. Protein concentrations in the lysates were determined by the BCA kit (Thermo Fisher Scientific). The cell lysate was treated with either control rabbit immunoglobulin G or primary antibodies and incubated overnight at 4 °C. On the following day, protein G magnetic beads were added to the supernatants and incubated for 2 h at 4 °C. Samples were then washed 3 times using a washing buffer. After washing, the beads were magnetically separated, resuspended, and boiled for 5 min at 95 °C. For every sample, total protein extracts (30 μg) were subjected to sodium dodecyl sulfate–polyacrylamide gel electrophoresis (SDS-PAGE) (Bio-Rad), and Western blotting was conducted. The antibodies used in this assay and their dilution ratios were listed in Table S2. The intensities of immunoreactive protein bands were visualized using chemiluminescence (Bio-Rad).

### RNA sequencing

Total RNA was extracted from cells using the Trizol reagent kit (Invitrogen, Carlsbad, CA, USA). The integrity of the RNA was evaluated using the Agilent 2100 Bioanalyzer (Agilent Technologies, Santa Clara, CA, USA). Eukaryotic mRNA was enriched using Oligo (dT) beads, and ribosomal RNA (rRNA) was removed by a Magnetic Kit (Epicentre, Madison, WI, USA). The enriched mRNA was fragmented using a fragmentation buffer and subsequently reverse-transcribed with random hexamers. The cDNA was then synthesized and purified using a Qiagen purification kit. The cDNA underwent end repair, addition of poly (A), and ligation. The ligated products were amplified via PCR and separated using agarose gel electrophoresis before being sequenced on the Illumina HiSeq2500 platform. Sequencing reads were filtered using Fastp (version 0.18.0), and reads aligning to rRNA were removed by Bowtie2 (version 2.2.8). Transcript assembly, gene expression quantification, and alignment to the reference genome were performed utilizing the filtered reads. The mapped reads were assembled using a reference-based approach with StringTie (version 1.3.1). DEGs were identified using DESeq2 software. Enrichment analysis of DEGs was conducted in R using the ClusterProfiler package (available at https://github.com/YuLab-SMU/clusterProfiler), which employed GO and KEGG.

### Culturing and transplanting mouse SSCs

Mouse SSCs with GFP (green fluorescence protein) were a gift from P. Zhang of Nanjing Medical University. Cell mixture was isolated from testicular tissues of mice with GFP at postnatal day 6 mice using a 2-step enzymatic digestion at 34 °C. Following centrifugation, the cells are resuspended in DMEM supplemented with 10% FBS, 1% penicillin, and 1% streptomycin. To enrich for SSCs, type A spermatogonia were separated using a differential plating technique. Subsequently, SSCs are isolated using magnetic-activated cell sorting (MACS) with SSC markers Thy1 and α6-integrin.

The isolated SSCs were plated onto mitotically inactivated mouse embryonic fibroblasts (MEFs) or extracellular matrix-coated dishes and maintained in a culture medium designed for SSC propagation. This medium typically included DMEM/F12 with knockout serum replacement (KSR), glutamine, β-mercaptoethanol, and necessary growth factors, e.g., GDNF, LIF, epidermal growth factor (EGF), and basic fibroblast growth factor (bFGF). SSCs were cultured in an incubator with humidified atmosphere at 34 °C and 5% CO_2_. When cells reached approximately 80% confluency, they were passaged using trypsin-EDTA or non-enzymatic dissociation methods to maintain SSC self-renewal and prevent differentiation.

Prior to transplantation, recipient mice were treated with 40 mg/kg busulfan to eliminate endogenous male germ cells. The cultured SSCs are dissociated enzymatically or mechanically, counted, and resuspended in a transplant buffer containing FBS. Using a micropipette under a stereomicroscope, mouse SSCs were transplanted into the seminiferous tubules of the recipient mouse testes. Two months after transplantation, the function of the transplanted SSCs was assessed by histological analysis to evaluate spermatogenesis and the potential production of offspring. Strict aseptic techniques were employed throughout these procedures to maintain cell viability and prevent contamination. The use of all experimental animals was approved by the Experimental Animal Welfare Ethics Committee of Central South University, and it was in accordance with the principles of experimental animal welfare ethics.

### Construction of *Apbb1* conditional knockout mice

The *Stra8*-GFP-Cre and Apbb1^flox/flox^ mice were obtained from Cyagen (Suzhou, China). The Apbb1^flox/flox^ mouse lines were developed using CRISPR-Cas9 technology, which involved the insertion of 2 loxP sites: one before exon 7 and another after exon 12 of the *Apbb1* gene. Genotyping of Apbb1^flox/flox^ mice was performed via PCR with tail genomic DNA. The primers used for PCR genotyping of Apbb1^flox/flox^ mice were listed in Table S1.

### WES of NOA patients

Genomic DNA was extracted from peripheral blood of NOA patients with a DNA extraction kit (QIAGEN) according to the manufacturer’s instructions. After determining concentrations and purity of DNA, the whole exome library was constructed and sequenced on the Illumina HiSeq 2000 or NovaSeq 6000 sequencing platforms (Illumina). The raw reads were aligned to GRCh37/hg19 with Burrows-Wheeler Aligner. Genetic variations, e.g., single-nucleotide variants (SNVs), deletions, and small insertions, were analyzed, and they were annotated and filtered by various public databases and silico tools, including 1000G, gnomAD, ExAC, SIFT, PolyPhen-2, and MutationTaster.

### Statistical analysis

The R programming language employed the dplyr package (https://dplyr.tidyverse.org) for data analysis. Each experiment was replicated a minimum of 3 times, and the results were presented as the mean ± SD. The *t* test was utilized to evaluate the differences in variances among the groups, and statistical significance was set at *P* < 0.05.

## Data Availability

The information produced by this research can be acquired from the corresponding authors upon a reasonable inquiry.
