# Supplementary material for: Essential Regulation of Spermatogonial Stem Cell Fate Decisions and Male Fertility by APBB1 via Interaction with KAT5 and GDF15 in Humans and Mice
Source: Research (Wash D C). 2025 Mar 27;8:0647. doi: 10.34133/research.0647 (PMC11948500; doi:10.34133/research.0647)
Supplement: Supplementary 1 — Figs. S1 to S5 Tables S1 to S5 [file research.0647.f1.zip › Supplementary information.docx]

**Supplementary information**

**Essential regulation of spermatogonial stem cell fate decisions and male fertility by APBB1 via interaction with KAT5 and GDF15 in humans and mice**

**Supplemental Data:**

**Figures S1-S5**

**Tables S1-S5**

**Supplemental Figures 1-5**

**Figure S1**

**
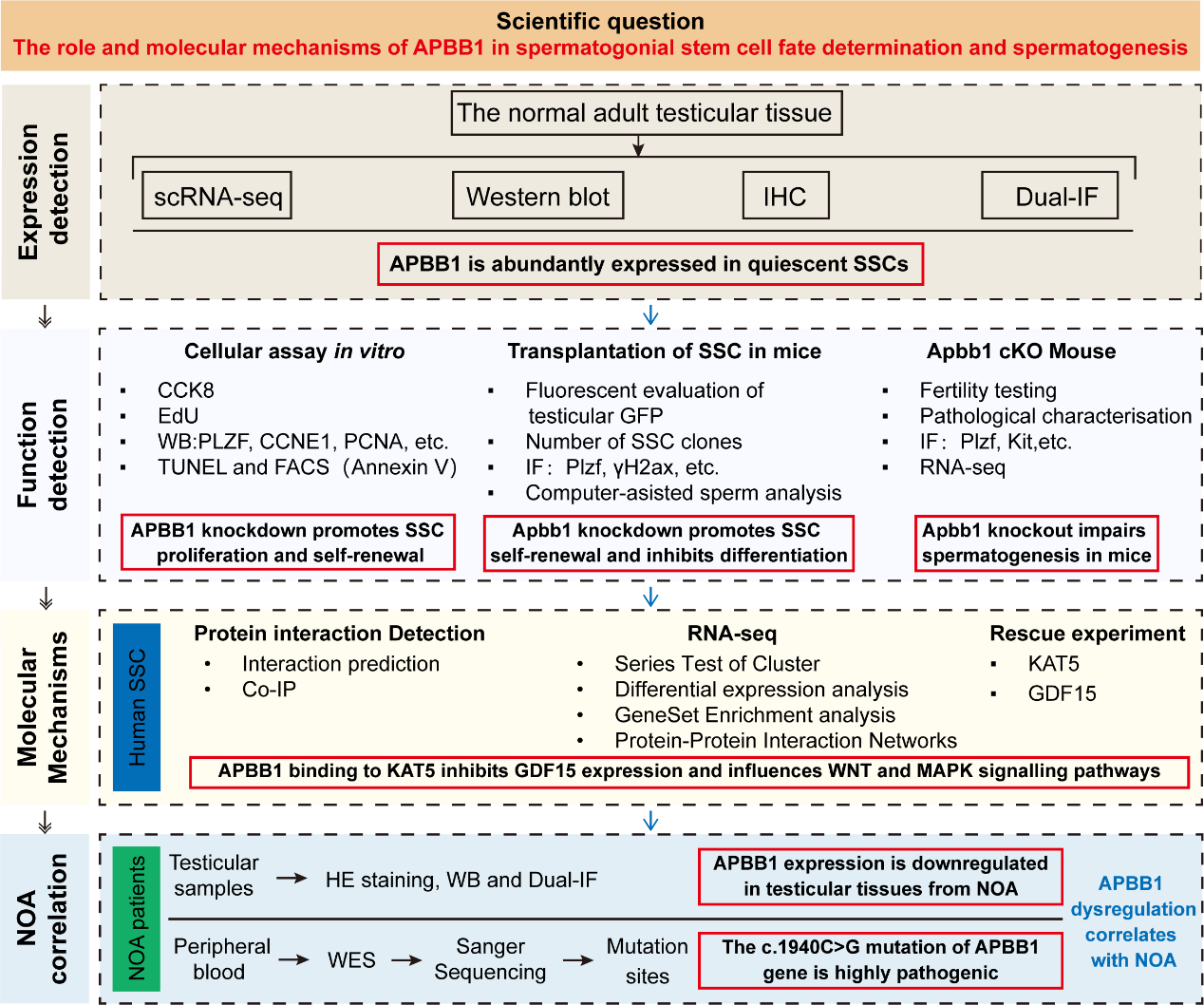
**

**Figure S1. Experimental design and procedures of this study**

**Figure S2**

**
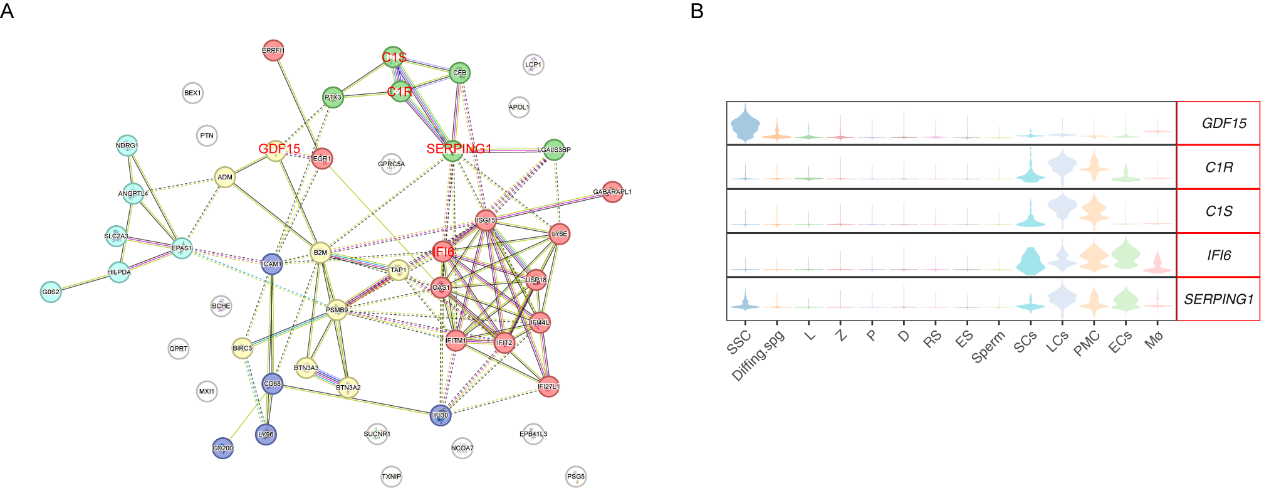
**

**Figure S2. PPI analysis for top 50 upregulated genes in RNA sequencing.** (A) PPI network of top 50 upregulated genes. (B) Expression of 5 hubgenes in scRNA profiles.

**Figure S3**

**
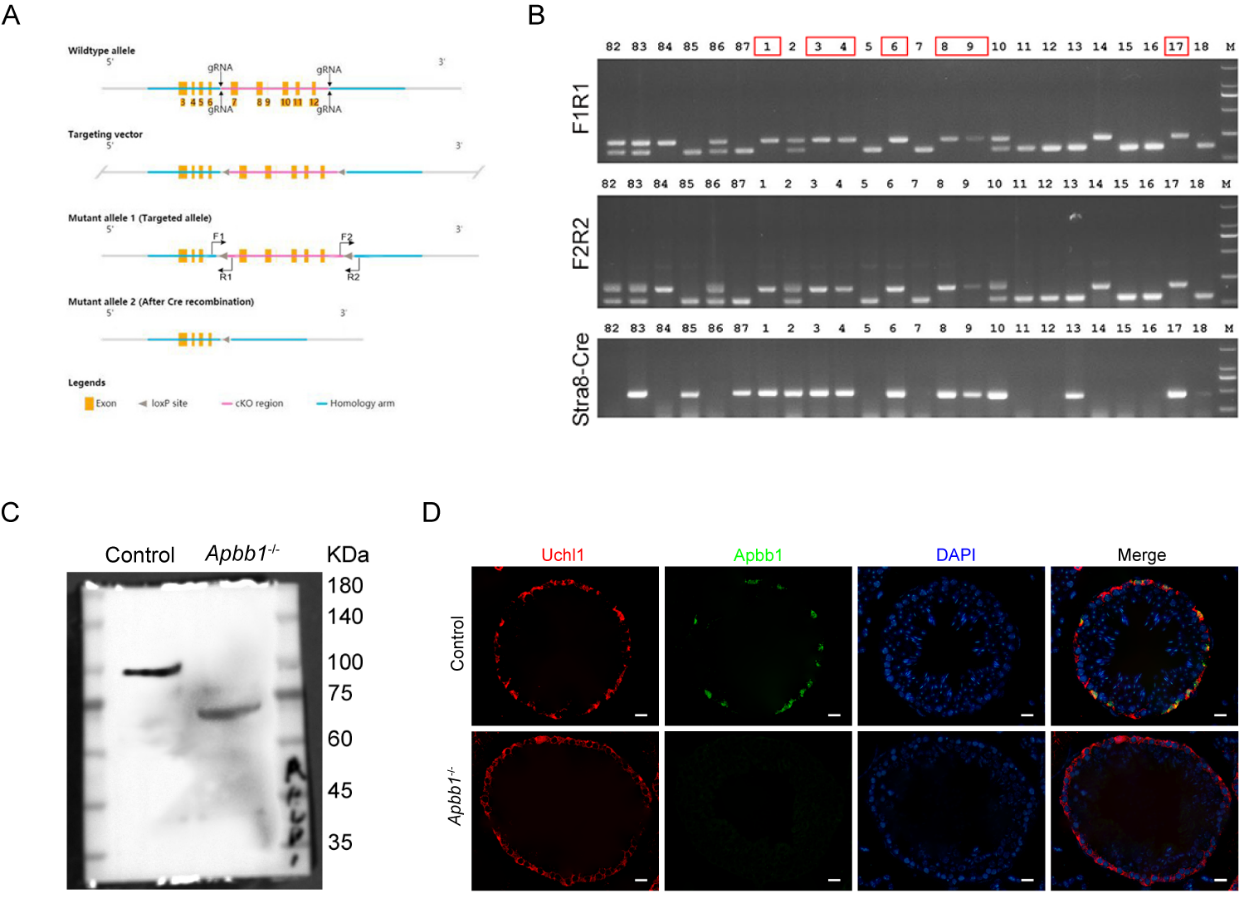
**

**Figure S3. Identification of Apbb1^-/-^ mice.** (A) Construction of Apbb1^floxed^ mice. LoxP sequences were inserted into the flanking regions of exons 7 to 12 AND designated as F1R1 and F2R2 sites. (B) Genotype of Apbb1^-/-^ mice by PCR. (C) Expression of Apbb1 in Apbb1^-/-^ mouse testis detected by Western blot. Deletion of exons 7 to 12 led to generation of truncated Apbb1 protein. (D) Immunofluorescence of Apbb1 localization in the Apbb1^-/-^ mouse testes. In normal mice, Apbb1 was co-localized with Uchl1, a marker for SSCs. In Apbb1^-/-^ mice, there was no significant change in Uchl1 localization, whereas Apbb1 staining was undetected in the seminiferous tubules of these mice. Scale bar: 20μm.

**Figure S4**

**
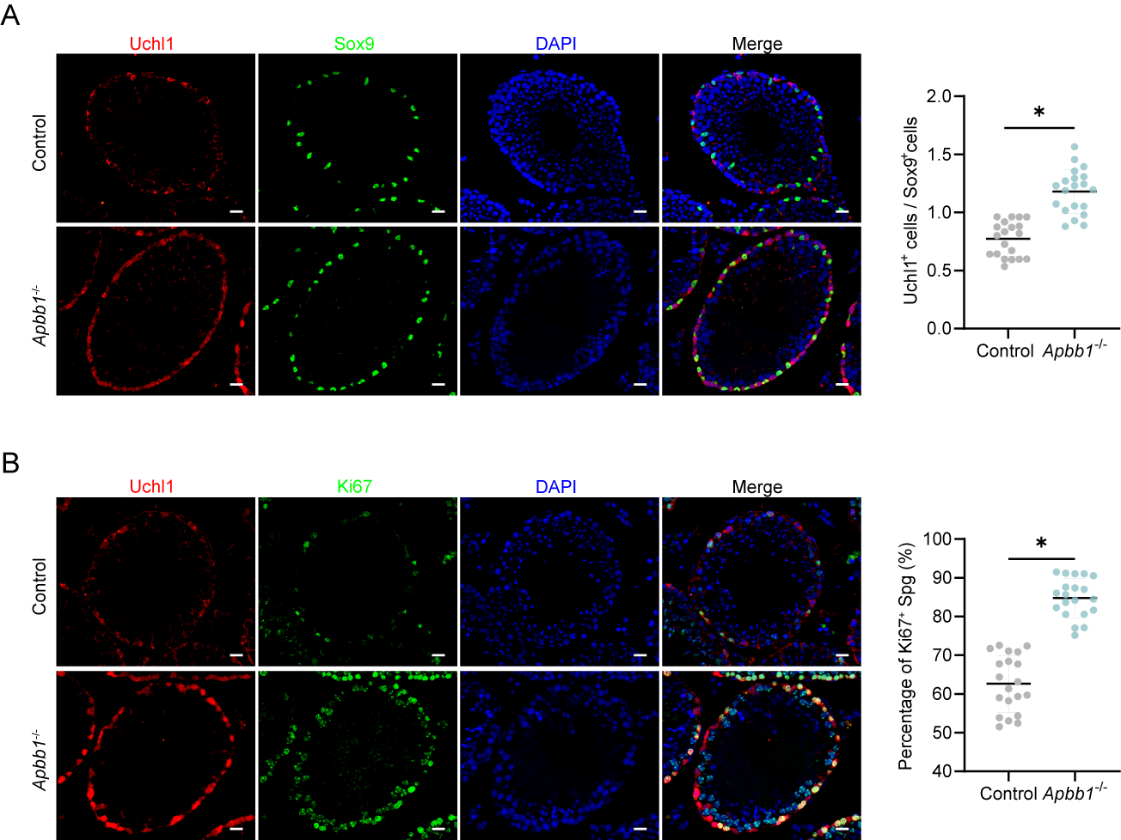
**

**Figure S4. Detection of the number and proliferation of SSCs with immunofluorescence in the testes of Apbb^-/-^ mice.** (A) The numbers of SSCs were detected by immunofluorescence. In comparison to the wild type control, there was a significant increase in the numbers of Uchl1-positive SSCs within the testes of Apbb1^-/-^ mice when normalized against Sertoli cells. (B) The proliferation analysis of SSCs using immunofluorescence. *Apbb1* deletion resulted in a significant upregulation of the percentages of proliferating spermatogonia (Ki67^+^). Scale bar: 20μm.

**Figure S5**


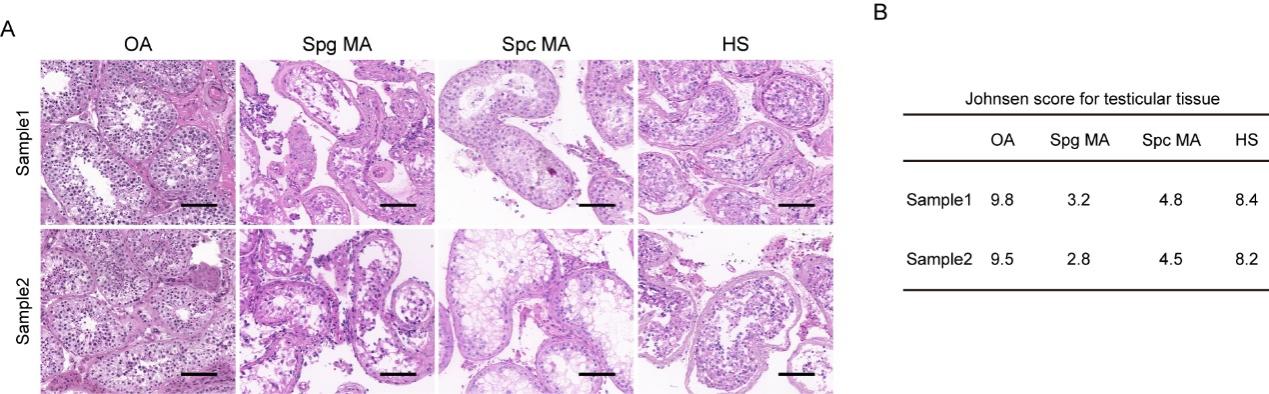


**Figure S5. H&E staining of testes from OA with normal spermatogenesis and NOA patients in Figure 10C and 10E.** (A) H&E staining of testes from OA with normal spermatogenesis and NOA patients, including maturation arrest at spermatogonia (Spg MA), maturation arrest at spermatocytes (Spc MA) and hypospermatogensis (HS). Scale bar: 50μm. (B) Johnsen scores for each testicular tissues.

**Supplemental Tables**

**Table S1. Primer sequences of genes for humans and mice**

| **Genes** | **Sequences** | **Genes** | **Sequences** |
| --- | --- | --- | --- |
| *APBB1* | **F:** GGAGGGGACGTTGACCTTC | *Apbb1* | **F:** AGGAGGCCCCAATGGAGTT |
|  | **R:** TTTTGTGGTAAGAGAGCTGACG |  | **R:** GCGCACAGCGAAACACTTG |
| *GDF15* | **F:** ACCTGCACCTGCGTATCTCT | *F1R1* | **F:** GATGTGTGTATGAGCTGAACTGTC |
|  | **R:** CGGACGAAGATTCTGCCAG |  | **R:** GATGTAAGGTCCCCAAACAAGAC |
| *SERPING1* | **F:** GGGATGCTTTGGTAGATTTCTCC | *F2R2* | **F:** GAGGGAGGCTTTCTTTCCCTATC |
|  | **R:** GAGGATGCTCTCCAGGTTTGT |  | **R:** TATGCTTACAGAGTCCTTCTCGG |
| *TXNIP* | **F:** GGTCTTTAACGACCCTGAAAAGG | *cre* | **F:** GATGGATTTCCGTCTCTGGTGTAG |
|  | **R:** ACACGAGTAACTTCACACACCT |  | **R:** CCCATTTAATCTCCTCCTTCTCCG |
| *PTHLH* | **F:** ATTTACGGCGACGATTCTTCC | *Ascl2* | **F:** AAGCACACCTTGACTGGTACG |
|  | **R:** GCTTGGAGTTAGGGGACACC |  | **R:** AAGTGGACGTTTGCACCTTCA |
| *HHIP* | **F:** TACACTTGCCGAGGCCATATT | *Nrtn* | **F:** GGGCTACACGTCGGATGAG |
|  | **R:** CCCACTCACAACCTCCTGAAT |  | **R:** CCAGGTCGTAGATGCGGATG |
| *PLAT* | **F:** AGCGAGCCAAGGTGTTTCAA | *Spock1* | **F:** AGCGAGCCAAGGTGTTTCAA |
|  | **R:** CTTCCCAGCAAATCCTTCGGG |  | **R:** CCTACTGGGGGCCTTCATC |
|  | **R:** ACAGTGTCAGGTTGTCTCGC | *Zxdb* | **F:** CCCTACCAGTGCGCGTTTT |
|  |  |  | **R:** ACACAGGAAGGGTCTCTCCC |
|  |  | *Gpr141* | **F:** TTCCTCTTGTGACCCTATACTGG |
|  |  |  | **R:** CACGAGGTTGATGACAGCCA |
|  |  | *Fbxo32* | **F:** CAGCTTCGTGAGCGACCTC |
|  |  |  | **R:** GGCAGTCGAGAAGTCCAGTC |

**Table S2. Antibodies applied in Western blots, immunofluorescence and immunoprecipitation (IP)**

| **Antibodies** | **Sources** | **Dilution** | **Incubation** |
| --- | --- | --- | --- |
| **Western blots** |  |  |  |
| APBB1 | Sigma cat#HPA038522 | 1:800 | 12h at 4℃ |
| Apbb1 | santaCruz cat#sc398389 | 1:1000 | 12h at 4℃ |
| PLZF | SantaCruz cat#sc28319 | 1:1000 | 12h at 4℃ |
| KAT5 | Abcam cat#ab300522 | 1:800 | 12h at 4℃ |
| CCND1 | Abcam cat#ab134175 | 1:1000 | 12h at 4℃ |
| PCNA | Abcam cat#ab29 | 1:500 | 12h at 4℃ |
| CCNE1 | Abcam cat#ab33911 | 1:1000 | 12h at 4℃ |
| ACTB | Promab cat#20270 | 1:2000 | 12h at 4℃ |
| GFRA1 | R&D cat#AF560 | 1:500 | 12h at 4℃ |
| THY1 | Abcam cat#ab92574 | 1:500 | 12h at 4℃ |
| GDF15 | Zenbio cat#382507 | 1:1000 | 12h at 4℃ |
| ERK1/2 | Promab cat#30014 | 1:1000 | 12h at 4℃ |
| p-ERK1/2 | Zenbio cat#R380698 | 1:1000 | 12h at 4℃ |
| MEK1/2 | Promab cat#P22715 | 1:1000 | 12h at 4℃ |
| p-MEK1 | Promab cat#P20128 | 1:1000 | 12h at 4℃ |
| β-catenin | Abcam cat#ab32572 | 1:1000 | 12h at 4℃ |
| **Immunofluorescence** |  |  |  |
| APBB1 | Sigma cat# HPA038522 | 1:50 | 16h at 4℃ |
| DDX4 | Abcam cat#ab27591 | 1:100 | 16h at 4℃ |
| GFRα1 | R&D cat#AF560 | 1:25 | 16h at 4℃ |
| PCNA | Abcam cat#ab29 | 1:50 | 16h at 4℃ |
| KIT | R&D cat#AF332 | 1:25 | 16h at 4℃ |
| PLZF | SantaCruz cat#sc28319 | 1:100 | 16h at 4℃ |
| GFP | Abcam cat#ab6556 | 1:100 | 16h at 4℃ |
| p-γH2AX | Sigma cat# 05636 | 1:200 | 16h at 4℃ |
| UCHL1 | Bio-rad cat#78631004 | 1:100 | 16h at 4℃ |
| Apbb1 | santaCruz cat#sc398389 | 1:25 | 16h at 4℃ |
| KAT5 | Abcam cat# ab300522 | 1:50 | 16h at 4℃ |
| SOX9 | Sigma cat#AB5535 | 1:100 | 16h at 4℃ |
| Ki67 | Abcam cat#ab15580 | 1:50 | 16h at 4℃ |
| **Immunoprecipitation** |  |  |  |
| APBB1 | Sigma cat#05758 | 1:20 | 12h at 4℃ |
| KAT5 | Abcam cat#ab300522 | 1:30 | 12h at 4℃ |
| Rabbit-IgG | Abcam cat#ab172730 | 1:30 | 12h at 4℃ |

**Table S4. Mutation distribution of *APBB1* gene by whole exome sequencing (WES) in 2,047 patients with NOA**

| **Genes** | **Transcripts** | **Site of mutation** | | **Nucleotide changes** | **Amino acid changes** | **Numbers** | **dbSNP** |
| --- | --- | --- | --- | --- | --- | --- | --- |
| *APBB1* | NM_001164 | exon14 | c.2108A>C | | p.K703T | 1 | rs2134029565 |
| *APBB1* | NM_001164 | exon5 | c.979A>G | | p.M327V | 1 | rs1800423 |
| *APBB1* | NM_001164 | exon12 | c.1742G>T | | p.S581I | 1 | rs757637279 |
| *APBB1* | NM_001164 | exon2 | c.486_488del | | p.162_163del | 2 | - |
| *APBB1* | NM_001164 | exon2 | c.486G>T | | p.E162D | 1 | [rs145320037](https://www.ncbi.nlm.nih.gov/snp/?term=rs145320037) |
| *APBB1* | NM_001164 | exon13 | c.1940C>G | | p.S647X | 1 | - |
| *APBB1* | NM_001164 | exon13 | c.1931_1932insA | | p.A644fs | 1 | - |
| *APBB1* | NM_001164 | exon7 | c.1234A>G | | p.M412V | 1 | rs201889999 |

**Table S5. Risk analysis of disease associated with mutation sites in the *APBB1* Gene**

| **Genes** | **SNPs** | **Nucleotide changes** | **Amino acid changes** | **Allelic genes** | **Cases in patients** | **Cases in control** |
| --- | --- | --- | --- | --- | --- | --- |
| *APBB1* | rs2134029565 | c.2108A>C | p.K703T | A | 2047 | - |
|  |  |  |  | C | 1 | - |
| *APBB1* | rs1800423 | c.979A>G | p.M327V | A | 2047 | 281002 |
|  |  |  |  | G | 1 | 1498 |
| *APBB1* | rs757637279 | c.1742G>T | p.S581I | G | 2047 | 194505 |
|  |  |  |  | T | 1 | 5 |
| *APBB1* | - | c.486_488del | p.162del | GGA | 2046 | - |
|  |  |  |  | - | 2 | - |
| *APBB1* | [rs145320037](https://www.ncbi.nlm.nih.gov/snp/?term=rs145320037) | c.486G>T | p.E162D | G | 2047 | - |
|  |  |  |  | T | 1 | - |
| *APBB1* | - | c.1940C>G | p.S647X | C | 2047 | - |
|  |  |  |  | G | 1 | - |
| *APBB1* | - | c.1931_1932insA | p.A644fs | - | 2047 | - |
|  |  |  |  | A | 1 | - |
| *APBB1* | rs201889999 | c.1234A>G | p.M412V | A | 2047 | 282675 |
|  |  |  |  | G | 1 | 81 |
